# Microfluidics for the biological analysis of atmospheric ice-nucleating particles: Perspectives and challenges

**DOI:** 10.1063/5.0236911

**Published:** 2025-02-27

**Authors:** Mark D. Tarn, Kirsty J. Shaw, Polly B. Foster, Jon S. West, Ian D. Johnston, Daniel K. McCluskey, Sally A. Peyman, Benjamin J. Murray

**Affiliations:** 1 School of Earth and Environment, University of Leeds, Leeds LS2 9JT, United Kingdom; 2 Faculty of Science and Engineering, Manchester Metropolitan University, Manchester M1 5GD, United Kingdom; 3 School of Physics and Astronomy, University of Leeds, Leeds LS2 9JT, United Kingdom; 4 Protecting Crops and Environment Department, Rothamsted Research, Harpenden AL5 2JQ, United Kingdom; 5 School of Physics, Engineering and Computer Science, University of Hertfordshire, College Lane, Hatfield AL10 9AB, United Kingdom; 6 Institute of Biological Chemistry, Biophysics and Bioengineering, Heriot-Watt University, Edinburgh EH14 4AS, United Kingdom

## Abstract

Atmospheric ice-nucleating particles (INPs) make up a vanishingly small proportion of atmospheric aerosol but are key to triggering the freezing of supercooled liquid water droplets, altering the lifetime and radiative properties of clouds and having a substantial impact on weather and climate. However, INPs are notoriously difficult to model due to a lack of information on their global sources, sinks, concentrations, and activity, necessitating the development of new instrumentation for quantifying and characterizing INPs in a rapid and automated manner. Microfluidic technology has been increasingly adopted by ice nucleation research groups in recent years as a means of performing droplet freezing analysis of INPs, enabling the measurement of hundreds or thousands of droplets per experiment at temperatures down to the homogeneous freezing of water. The potential for microfluidics extends far beyond this, with an entire toolbox of bioanalytical separation and detection techniques developed over 30 years for medical applications. Such methods could easily be adapted to biological and biogenic INP analysis to revolutionize the field, for example, in the identification and quantification of ice-nucleating bacteria and fungi. Combined with miniaturized sampling techniques, we can envisage the development and deployment of microfluidic sample-to-answer platforms for automated, user-friendly sampling and analysis of biological INPs in the field that would enable a greater understanding of their global and seasonal activity. Here, we review the various components that such a platform would incorporate to highlight the feasibility, and the challenges, of such an endeavor, from sampling and droplet freezing assays to separations and bioanalysis.

## I. INTRODUCTION

“Historically, the measurement of ice nucleating activity has been found to be stubbornly difficult. Ice nucleation is sensitive to a large number of complex variables, so that the requirement that the measurements reflect the reaction of the nuclei to the state of those variables in natural clouds, is indeed a demanding one.”

This statement by Gabor Vali was written 50 years ago in a report on *The 3rd International Workshop on the Measurement of Ice Nuclei* in 1975^1,2^ regarding our understanding at the time of what we now call ice-nucleating particles (INPs). INPs are a rare aerosol particle type that can trigger freezing in supercooled cloud water droplets and so drastically alter the radiative properties and lifetime of clouds,^3,4^ in turn influencing weather and climate.^5–7^ While there have been many great strides and findings made in both fundamental and atmospheric ice nucleation research in the decades since, in some ways the same statement could just as easily be made today.

We now have a far greater overview of the types of particles that can nucleate ice in the atmosphere,^3^ their influence on cloud systems,^8,9^ and a greater understanding of their sources and concentrations via a number of global field campaigns (see the Ice Nucleation DataBase, INDB, that collates data from 50 years of INP campaigns: https://www.bacchus-env.eu/in/).^10,11^

Desert dusts^12–14^ and sea spray aerosols (SSAs)^13,15,16^ have long been known as two of the most important INPs in the atmosphere^17–19^ and so are typically used to represent INPs in global aerosol models.^13,17–20^ K-feldspar mineral dust tends to dominate the atmospheric INP population where present.^17,21–23^ SSAs comprising biogenic and organic materials,^13,15,16,24–28^ including bacteria, viruses, phytoplankton, and diatom fragments,^24–27,29–33^ aerosolized by wave breaking and bubble-bursting processes,^24,34^ represent less active INPs that can nonetheless become important in remote marine environments.^18,24,28,34^

However, data from a number of field campaigns have demonstrated that there are “missing sources” of high temperature INPs in the models,^35,20,36^ i.e., INPs that trigger freezing at warmer temperatures (closer to 0 °C) than mineral dusts and which may be of terrestrial biological origin, e.g., fertile soils and associated microorganisms.^19,20,36–51^ In particular, mineral dusts are believed to dominate the INP population at temperatures below ∼−18 to −20 °C when present (outside of remote marine environments), while INPs of biological and biogenic (i.e., materials produced by organisms) origin are believed to be important at temperatures warmer than around −18 to −15 °C, often presenting as a “biological hump” in INP temperature spectra during field measurements.^12,37,39,40,52–54^

Bioaerosols are believed to dominate ice-nucleating activity at warmer temperatures, depending on whether they are in high enough concentrations to compete with other sources such as mineral dust,^55^ and are a key uncertainty in the predictability of INPs in models.^37^ Indeed, ice-nucleating bacteria have been found in the atmosphere^26,55–58^ and in rainwater,^59–61^ snow,^59,60,62–64^ hail,^63,65^ sleet,^60^ and cloud water.^44,66^ Likewise, fungal spore INPs and pollen-based INPs have also been found in atmospheric samples such as rainwater and cloud ice crystals,^56,67–69^ and both fungal spores^26,55,56,70–73^ and pollen (and their contents)^26,57,74–76^ can be emitted into the atmosphere. Biological INPs can be found in (or associated with) terrestrial sources such as plants and trees (including pollen, bark, leaves, branches, and stems),^77–81^ decaying leaf litter,^48,49,82–84^ fertile and agricultural soils,^42,53,58,85–87^ fungi,^88^ crops,^80^ fruit^89,90^ and vegetables,^91^ moss,^92,93^ liverworts,^93^ and lichen on trees, rocks, and soils.^94–97^

While ice-nucleating biological material can comprise intact cells or grains, they can also be present as cell fragments or can produce or contain ice-nucleating macromolecules (INMs).^98–100^ Some species of bacteria and fungi produce ice-nucleating proteins,^101–105^ while pollen contains subpollen particle (SPP) INMs believed to be polysaccharides,^74,99,100,106,107^ which may also be a form of INMs in fungi.^103^ Further, INMs can be transported into the atmosphere with, and when attached to, dust and soil particles.^13,14,98,108,109^

Many lichens have been identified as excellent sources of warm-temperature INPs,^94–97^ and tree-borne lichens may be important in boreal forest regions particularly when the ground is otherwise snow-covered.^50,94^ Some viruses^110,111^ and archaea^112^ are also ice nucleation active, though it is not clear that they are present in sufficient concentrations to compete with other INPs. Likewise, cold-tolerant tardigrades^113^ and insects^114–116^ can contain exogenous (i.e., in the gut or body) or endogenous (i.e., in the hemolymph fluid and muscle) ice-nucleating agents,^117–121^ alongside ice-binding proteins^122^ (including antifreeze and glycoproteins),^123,124^ to survive in freezing conditions, but their impact on the atmosphere may be low, if at all, due to their relatively low abundance in the atmosphere.

A comprehensive list of known biogenic INPs is provided in Table I in the Appendix. The reader is also directed to more focused reviews of biological ice-nucleating particles in the atmosphere,^3,55,125^ including specialized reviews of ice-nucleating pollen^74^ and bacteria.^13,43,58,126^ However, it must be noted that levels of ice-nucleating activity can vary within the same species. The best example of this is the most well-known ice-nucleating bacteria, *Pseudomonas syringae (P. syringae)*,^127^ which has a number of strains that are deemed “not ice active,”^128–130^ although the minimum temperature at which a species is dubbed “non-active” may be limited by the experimental technique rather than the sample having no activity.

**TABLE I.** List of known biological ice-nucleating particles. Note that many (if not all) species also contain strains that may not be ice-nucleating or have varying ice-nucleating activity.

Organism	Notes	References
**Bacteria**		
*Bacillus* sp.	Gram-positive soil bacteria	61
*Brevibacterium* sp.	Gram-positive soil-based actinobacteria	61
*Cellulosimicrobium* sp.	Gram-positive human pathogen	61
*Cupriavidus pauculus*	Waterborne human pathogen	542
*Erwinia ananas*	Contains *inaA* gene^148^	148,543
*Erwinia stewartii*	Plant pathogen	544
*Exiguobacterium* sp.	Extremophile	545
*Flavobacterium* sp.	Soil and fresh water bacteria	545
*Idiomarina* sp.	Marine bacteria	61
*Lysinibacillus* sp.	Gram-positive bacteria. Freezing rain sample, source: Virginia, USA	140
*Lysinibacillus parviboronicapiens*	Gram-positive bacteria. Freezing rain sample, source: Virginia, USA	140,141
*Paenibacillus* sp.	Gram-positive, endospore-forming bacteria	61
*Pantoea* sp.	Opportunistic human pathogen	61
*Pantoea agglomerans* (formerly *Erwinia herbicola*)	Contains *inaE* (*iceE*) gene^154^	63,546–548
*Pantoea ananatis* (formerly *Erwinia uredovora*)	Plant pathogen, contains *inaA*^149^ and *inaU*^151^ genes	63,549
*Phormidium* cf. *attenuatum*	Source: Antarctic soil, cyanobacterium	550
*Phormidium scottii*	Source: Antarctic soil, cyanobacterium	550
*Planococcus* sp.	Gram-positive bacteria	61
*Prochlorococcus* sp.	Marine bacterium	551
*Pseudomonas* sp.	Plant pathogen	56,64,66,552
*Pseudomonas aeruginosa*	Plant and animal pathogen	217
*Pseudomonas antarctica*	Isolated from sand, source: Ross Island, Antarctica	553,554
*Pseudomonas auricularis* ^a^	Snow sample, source: Greece	63
*Pseudomonas borealis*	Contains *inaPb* gene^153^	375,376,555
*Pseudomonas fluorescens*	Contains *inaW* gene^150^	63,547,553,556,557
*Pseudomonas poae* ^a^	Non-pathogenic	63
*Pseudomonas putida*	Soil bacterium	63,558
*Pseudomonas syringae*	Plant pathogen, contains *inaZ*,^101^ *inaC*,^144^ *inaK*,^145^ *inaV*,^146^ and *inaQ*^147^ genes	63,66,88,127,129,547,553,557,559–562
*Pseudomonas syringae* as Snomax®	Sterilized and lyophilized form of *P. syringae*^563^	35,89,99,201,203,204,206,317,321,369,561,564–567
*Pseudomonas syringae* pv. *Coronafaciens*	Plant pathogen	130
*Pseudomonas syringae* pv. *lachrymans*	Plant pathogen	130
*Pseudomonas syringae* pv. *Pisi*	Plant pathogen	130
*Pseudomonas viridiflava*	Plant pathogen	63,90,553,568–570
*Pseudophormidium* sp.	Source: Antarctic soil, cyanobacterium	550
*Pseudoxanthomonas* sp.	Plant pathogen	66,571
*Psychrobacter* sp.	Human pathogen	61,545
*Sphingomonas* sp.	Human pathogen	545
*Stenotrophomonas* sp.	Plant and animal pathogen, antibiotic resistant	140
*Vibrio harveyi*	Marine bacterium	572
*Xanthomonas* sp.	Plant pathogen	66,561
*Xanthomonas campestris*	Plant pathogen	63,66,571
*Xanthomonas campestris* pv. *raphani*	Plant pathogen	63
*Xanthomonas campestris* pv. *Translucens*	Plant pathogen, contains *inaX* gene^154^	63,556
**Fungi**		
*Aureobasidium* sp.	Yeast	573
*Cladosporium* spores	Common mold, plant pathogen and allergen	574
*Cryptococcus* sp.	Cryptococcaceae (contains yeasts and filamentous forms)	61
*Fusarium* sp.	Plant and animal pathogen	118,119
*Fusarium acuminatum*	Plant pathogen	88,103,139,575
*Fusarium armeniacum*	Plant pathogen	103
*Fusarium avenaceum*	Plant pathogen	88,98,103,108,128,201,575,576
*Fusarium begoniae*	Plant pathogen	103
*Fusarium concentricum*	Plant pathogen	103
*Fusarium langsethiae*	Cereal pathogen	103
*Fusarium oxysporum*	Plant pathogen	577
*Fusarium sporotrichioides*	Plant pathogen	56
*Fusarium tricinictum*	Plant pathogen	103
*Isaria farinosa*	Entomopathogenic (insect pathogen)	56,100,103
*Metschikowia* sp.	Yeast	61
*Mortierella alpina*	Soil fungi	100,103,136
*Puccinia* sp.	Rust fungi	103,578
*Puccinia allii*	Rust fungi	578
*Puccinia aristidae*	Rust fungi	578
*Puccinia graminis* f. sp. *Tritici*	Rust fungi	578
*Puccinia lagenophorae*	Rust fungi	578
*Puccinia striiformis*	Rust fungi	578
*Puccinia triticina*	Rust fungi	578
*Sarocladium implicatum* (formerly named *Acremonium implicatum*)	Wheat fungi	56,100,103
**Pollen**		
*Abies balsamea*	Balsam fir	107
*Acer negundo*	Manitoba maple	579
*Acer pseudoplatanus*	Sycamore maple	107,579
*Agrostis alba*	Redtop grass	580
*Agrostis gigantea*	Redtop	99
*Alnus glutinosa*	Common European alder	106,107
*Alnus incana*	Grey alder	579,580
*Amaranthus hybridus*	Smooth pigweed	107
*Ambrosia artemisiifolia*	Ragweed	99
*Ambrosia trifida*	Giant ragweed	581
*Araucaria araucana*	Monkey puzzle tree	107
*Artemisia absinthium*	Wormwood	99
*Arundo formosana*	Taiwanese reed grass	107
*Betula alba*	White birch pollen	582,583
*Betula alleghaniensis*	Swamp birch	107
*Betula* x *caerulea*	Hybrid birch	107
*Betula ermanii*	Erman's birch	107
*Betula fontinalis occidentalis*	Water birch	584
*Betula pendula*	Silver birch	79,98–100,106,107,201,203,321,579,585–587
*Betula utilis* subsp. *Jacquemontii*	Himalayan birch	107
*Camellia reticulata*	Camellia species	107
*Camellia saluenensis*	Camellia species	107
*Carpinus betulus*	European hornbeam	107,586
*Carpinus cordata*	Heartleaf hornbeam	107
*Cedrus atlantica*	Atlas cedar	107
*Cedrus atlantica* f. *glauca*	Blue atlas cedar	107
*Cedrus deodara*	Deodar cedar	107
*Cestrum fasciculatum*	Early jessamine/red cestrum	107
*Clerodendrum speciosissimum*	Java glorybower	107
*Corylus avellana*	Common hazel	99,107
*Crocus vernus*	Spring crocus/giant crocus	107
*Cupressus arizonica*	Arizona cypress	579
*Cupressus sempervirens*	Mediterranean cypress	107
*Cynosurus cristatus*	Crested dog's-tail	107
*Dactylis glomerata*	Cat grass	107,582,583
*Encephalartos equatorialis*	Cycad species found in Uganda	107
*Erica multiflora*	Mediterranean heath	107
*Fraxinus pennsylvanica*	Red ash	579
*Juniperus chinensis pfizeriana*	Pfitzer juniper	99
*Juniperus communis*	Common juniper	99,321,579
*Hedychium coronarium*	White ginger lily	107
*Helianthus annuus*	Common sunflower	107
*Hordeum vulgare*	Barley	107
*Hymenocallis littoralis*	Beach spider lily	107
*Juglans regia*	English walnut	107
*Lolium* sp.	Ryegrass	581
*Morus rubra*	Red mulberry	579
*Musa rubra*	Wild banana	107
*Narcissus papyraceus* subsp. *polyanthos*	Paperwhite	107
*Nymphaea* “Kew's Stowaway Blues”	Tropical day blooming water lily	107
*Ostrya carpinifolia*	Hop hornbeam	107
*Picea abies*	Norway spruce	107
*Picea brachytyla*	Sargent spruce	107
*Pilgerodendron uviferum*	Conifer	107
*Pinus contorta* var. *contorta*	Shore pine	107
*Pinus coulteri*	Coulter pine	107
*Pinus halepensis*	Aleppo pine	107
*Pinus mugo*	Dwarf mountain pine	107
*Pinus ponderosa*	Ponderosa pine	107
*Pinus sylvestris*	Scots pine	99,106,582,583
*Plantago lanceolata*	Ribwort plantain	107
*Platanus orientalis*	Plane tree	99
*Poa pratensis*	Kentucky blue pollen	580
*Populus nigra*	Black poplar	580
*Populus nigra* v. *italica*	Lombardy poplar	579
*Quercus rubra*	Red oak	579,582,583
*Quercus suber*	Cork oak	107
*Quercus velutina*	Black oak	579
*Quercus virginiana*	Live oak	581
*Salix caprea*	Goat willow	99
*Sambucus nigra*	Common elder	107
*Sequoiadendron giganteum*	Giant sequoia	107
*Spathiphyllum wallisii*	Peace lily	107
*Taxus baccata*	Common yew	99,107
*Triticum aestivum*	Common wheat	107
*Thuja occidentalis*	Northern whitecedar	99
*Thuja orientalis*	Chinese Arborvitae	99
*Urtica dioica*	Common (stinging) nettle	99
*Zea mays*	Corn	99
**Moss**		
*Andrae rothui*	In the form of leaf material	93
*Anthoceros punctatus*	In the form of leaf material	93
*Atrichum undulatum*	In the form of leaf material	93
*Aulacomnium turgidum*	In the form of leaf material	93
*Dichodontium palustre*	In the form of leaf material	93
*Dicranella palustris*	In the form of leaf material	93
*Homalothecium sericeum*	In the form of leaf material	93
*Hypnum cupressiforme*	In the form of leaf material	93
*Orthotrichium anomalum*	In the form of leaf material	93
*Orthotrichum diaphanum*	In the form of leaf material	93
*Polytrichum commune*	Moss spores;^92^ leaf material^93^	92,93
*Polytrichum juniperinum*	In the form of leaf material	93
*Racomitrium lanuginosum*	In the form of leaf material	93
*Sphagnum cuspidatum*	In the form of leaf material	93
*Sphagnum palustre*	In the form of leaf material	93
*Syntrichia latifolia*	In the form of leaf material	93
*Tortula muralis*	In the form of leaf material	93
**Phytoplankton**		
*Apocalathium malmogiense*	Dinoflagellate	33
*Ascophyllum nodosum*	Brown algae	588
*Botrydiopsis* cf. *eriensis*	Source: Antarctic soil, green algae	550
*Bracteacoccus* cf. *minor*	Source: Antarctic soil, green algae	550
*Bumilleria* sp.	Source: Antarctic soil, yellow-green algae	550
*Chlamydomonas* sp.	Source: Antarctic soil, green algae	550
*Chlamydomonas* cf. *nivalis*^b^	Snow algae, Alga of the Year 2019	589
*Chlamydomonas reinhardtii*	Unicellular green alga	589
*Chlorella minutissima*	Source: Antarctic soil, green algae	550
*Chlorella vulgaris*	Source: Antarctic soil, green algae	550
*Chlorococcum* sp.	Source: Antarctic soil, green algae	550
*Chloromonas nivalis*	Snow algae	589
*Chlorophyta*-*Chlorophyceae*	Green algae	33
*Chlorophyta*-*Trebouxiophyceae*	Green algae	33
*Coccomyxa* sp.	Source: Antarctic soil, green algae	550
*Desmococcus olivaceus*	Green algae	33
*Dictyosphaerium chlorelloides*	Source: Antarctic soil, green algae	550
*Elliptochloris subsphaerica*	Source: Antarctic soil, green algae	550
*Emiliania huxleyi*	Coccolithopore, Alga of the Year 2009	572,590
*Fernandinella alpine*	Source: Antarctic soil, snow algae	550
*Fragilariopsis cylindrus* ^b^	Sea ice diatom, Alga of the Year 2011^591^	32
*Fucus serratus*	Brown algae	588
*Fucus spiralis*	Brown algae	588
*Fucus vesiculosus*	Brown algae	588
*Gonyostomum semen*	Algae	33
*Interfilum paradoxum*	Source: Antarctic soil, green algae	550
*Heterocapsa niei* (formerly *Cachonmia niei*)	Dinoflagellate	27,592
*Klebsormidium flaccidium* ^b^	Source: Antarctic soil, green algae, Alga of the Year 2018	550
*Laminaria digitata* ^b^	Brown algae, Alga of the Year 2007 (*Laminaria* sp.)	588
*Laminaria saccharina* ^b^	Brown algae, Alga of the Year 2007 (*Laminaria* sp.)	588
*Mastocarpus stellatus*	Red algae	588
*Melosira arctica* ^b^	Algae, Alga of the Year 2016	593
*Microcystis* sp.^a^	Cyanobacteria	33
*Myrmecia irregularis*	Source: Antarctic soil, green algae, associated with lichen	550
*Nannochloris atomus*	Green algae	63,572,590
*Ochromonus Danica*	Golden algae	27
*Palmaria palmata*	Red algae	588
*Pelvetia canaliculata*	Brown algae	588
*Peridinium aciculiferum*	Brown algae	33
*Phaeocystis* sp.	Algae	542
*Phormidium* cf. *attenuatum*	Source: Antarctic soil, cyanobacterium	550
*Phormidium scottii*	Source: Antarctic soil, cyanobacterium	550
*Porphyridium aerugineum*	Red algae	27
*Polarella glacialis*	Dinoflagellate	33
*Prochlorococcus marinus*	Cyanobacterium	63
*Prasiola crispa*	Source: Antarctic soil, green algae	550
*Pseudococcomyxa simplex*	Source: Antarctic soil, green algae	550
*Pseudophormidium* sp.	Source: Antarctic soil, cyanobacterium	550
*Rhopalocystis cucumis*	Source: Antarctic soil, green algae	550
*Schizochlamydella minutissima*	Source: Antarctic soil, golden algae	550
*Scotiellopsis* sp.	Source: Antarctic soil, green algae	550
*Scotiellopsis terrestris*	Source: Antarctic soil, unicellular green algae	550
*Stichococcus bacillaris*	Green algae	33,550
*Stramenopiles*-*Xanthophyceae*	Yellow-green algae	33
*Synechococcus elongatus*	Cyanobacterium	30
*Tetracystis vinatzeri*	Green algae	33
*Thalassiosira pseudonana*	Diatom	31,34,572,594
*Thalassiosira weissflogii*	Diatom	30
*Trebouxia asymmetrica*	Lichen symbiotic algae	589
*Trebouxia decolorans*	Green algae	33
*Trebouxia erici*	Lichen symbiotic algae	589
*Trebouxia glomerata*	Lichen symbiotic algae	589
*Xanthonema debile*	Source: Antarctic soil, yellow-green algae	550
**Lichen**		
*Acarospora* sp.	Source: New Mexico, USA; Colorado, USA	95
*Alectoria sarmentosa*	Source: Alaska	97
*Aspicilia contorta*	Source: UK	96
*Bryocaulon divergens*	Source: Alaska	97
*Bryoria* sp.	Sources: Alaska,^97^ Hyytiälä, Finland^94^	94,97
*Bryoria fuscescens*	Source: Alaska	97
*Buellia frigida*	Source: Antarctica	96
*Caloplaca* sp.	Source: UK	96
*Candelariella vitellina*	Source: UK	96
*Cetrariella delisei*	Source: Norway	96
*Cladonia* sp.	Source: UK	96
*Cladonia chlorophaea*	Source: UK	96
*Cladonia coniocraea*	Source: UK	96
*Cladonia cristatella*	Source: Alaska	97
*Cladonia macilenta*	Source: Alaska	97
*Cladina portentosa*	Source: Alaska	97
*Cladonia pyxidata*	Source: UK	96
*Cladonia rangiferina*	Source: Norway	96
*Cladonia squamosa*	Source: Alaska	97
*Clauzadea immersa*	Source: UK	96
*Dactylina arctica*	Source: Alaska	97
*Evernia prunastri*	Sources: UK,^96^ Alaska,^97^ Hyytiälä, Finland^94^	94,96,97
*Farnoldia jurana*	Source: UK	96
*Flavocetraria nivalis*	Sources: Norway,^96^ Alaska^97^	96,97
*Hypogymnia enteromorpha*	Source: Alaska	97
*Hypogymnia physodes*	Source: Hyytiälä, Finland	94
*Imshaugia aleurites*	Source: UK	96
*Lasallia pustulata*	Source: UK	96
*Lecanora gangaleoides*	Source: UK	96
*Lepraria* sp.	Source: UK	96
*Leptogium* sp.	Sources: UK,^96^ New Mexico, USA^95^	95,96
*Letharia* sp.	Source: California, USA	95,595
*Lobaria oregana*	Source: Alaska	97
*Lobaria pulmonaria*	Source: Alaska	97
*Nephroma arcticum*	Source: Norway	96
*Parmelia omphalodes*	Source: UK	96
*Parmelia saxatilis*	Sources: UK, Faroes Islands	96
*Parmelia sulcata*	Source: Alaska	97
*Parmotrema perlatum*	Source: UK	96
*Pelitgera* sp.	Source: New Mexico, USA	95
*Peltigera britannica*	Source: Alaska	97
*Peltigera neopolydactyla*	Source: Alaska	97
*Pertusaria hemisphaerica*	Source: UK	96
*Pertusaria hymenea*	Source: UK	96
*Physcia adscendens*	Source: UK	96
*Physcia tenella*	Source: UK	96
*Platismatia* sp.	Source: New Mexico, USA	95,595
*Platismatia glauca*	Source: Hyytiälä, Finland	94
*Platismatia herrei*	Source: Alaska	97
*Platismatia norvegica*	Source: Alaska	97
*Porpidia* sp.	Sources: Alaska,^97^ UK^96^	96,97
*Protoblastenia incrustans*	Source: UK	96
*Psora decipiens*	Source: New Mexico, USA	95
*Ramalina subfarinacea*	Source: UK	96
*Rhizocarpon geographicum*	Source: UK	96
*Rhizoplaca chrysoleuca*	Source: New Mexico, USA	95,595,596
*Solorina crocea*	Source: Norway	96
*Sphaerophorus globosus*	Source: Alaska	97
*Stereocaulon* sp.	Source: Alaska	97
*Stereocaulon alpina*	Source: Norway	96
*Stereocaulon alpinum*	Source: Alaska	97
*Stereocaulon evolutum*	Source: UK	96
*Stereocaulon vesuvianum*	Source: UK	96
*Sticta fulignosa*	Source: Alaska	97
*Thamnolia tundrae*	Source: Alaska	97
*Thamnolia vermicularis*	Source: Norway	96
*Usnea* sp.	Sources: UK,^96^ New Mexico, USA^95,595^	95,96,595
*Usnea longissima*	Source: Alaska	97
*Usnea wirthii*	Source: Alaska	97
*Xanthoparmelia* sp.	Source: New Mexico, USA	95,595
*Xanthoparmelia cumberlandia*	Source: Alaska	97
*Xanthoria calcicola*	Source: UK	96
*Xanthoria candelaria*	Source: UK	96
*Xanthora elegans*	Source: New Mexico, USA	95
*Xanthoria parietina*	Source: UK	96
**Liverworts**		
*Aneura pinguis*	In the form of leaf material	93
*Eucalypta streptocarpa*	In the form of leaf material	93
*Fissenden bryoides*	In the form of leaf material	93
*Frullania tamarisci*	In the form of leaf material	93
*Lepidozia reptans*	In the form of leaf material	93
*Lunularia cruciata*	In the form of leaf material	93
*Metzgeria temperata*	In the form of leaf material	93
*Plagiochila porelloides*	In the form of leaf material	93
**Viruses**		
His1	Bacteriophage	110
HRPV6	Bacteriophage	110
Phi6	Bacteriophage	110
Phi8	Bacteriophage	110
Phi12	Bacteriophage	110
Phi13	Bacteriophage	110
Phi2954	Bacteriophage	110
PhiX174	Bacteriophage	110
PRD1	Bacteriophage	110
Tobacco mosaic	Plant virus	111
**Archaea**		
*Halococcus morrhuae*	Prokaryotic organism	112
*Haloferax sulfurifontis*	Prokaryotic organism	112
**Tardigrades**		
*Adorybiotus coronifer*		113
*Milnesium tardigradum*		597

^a^ Noted as “possibly ice-nucleating” by the publication's authors.

^b^ The Alga of the Year is selected by the Phycology Section of the German Botanical Society (German Society for Plant Sciences): https://www.dbg-phykologie.de/en/alga-of-the-year.

There is a need to address the missing biological sources in aerosol and climate models,^20,36,125^ as well as to characterize the ice-nucleating properties of SSAs, but thus far the tools to achieve this have been limited,^125^ either in (i) specificity, (ii) sensitivity, or (iii) lack of broad use throughout the community due to complexity or cost. Hence, new instrumentation is required to meet these requirements, as highlighted by several recent reviews on the status and future of atmospheric ice nucleation research.^51,125,131–133^

A common technique for assessing the presence of biological ice-nucleating entities is the simple “heat test” for proteinaceous INPs,^13,59,134^ in which an aqueous sample is heated to denature the ice-nucleating proteins and results in a lower ice nucleation activity compared to the original sample. However, other non-proteinaceous materials (such as quartz) can also lose activity upon such a treatment, hence there are many caveats to consider when interpreting these test results.^134^ Other treatments include hydrogen peroxide to test for organic INPs and filtration for INMs, among others,^125^ but each relies on a comparative decrease in activity to investigate one broad class of INP materials. Multiple tests per sample would, therefore, be required to address each class, a very impractical strategy when conducting field campaigns.

Other methodologies such as genomic analysis of bacterial [via 16S ribosomal RNA (rRNA) sequencing]^42,135^ and fungal communities [via internal transcribed spacer (ITS) region sequencing],^135,136^ or scanning electron microscopy with energy dispersive x-ray spectroscopy (SEM-EDS),^137,138^ can provide an overview of the aerosol populations in a sample, but not which aerosols are ice nucleation active.

One of the most powerful bacterial INP detection methods available is quantitative polymerase chain reaction (qPCR) that allows for the identification and quantification of the *ina* gene, which encodes the ice-nucleating proteins of certain Gram-negative bacteria.^42,63^ However, this requires expertise and instrumentation that is not commonplace in ice nucleation research groups, while INPs containing the known *ina* genes may only comprise a small portion of the biological INP population.^42^ For example, while bacterial ice nucleation is caused by *ina* encoded proteins on the outer cell membrane, fungal ice nucleation is enabled by aggregation of extracellular proteins encoded by different genes that are largely, as yet, unidentified.^139^ Further, it was recently demonstrated that there are Gram-positive bacteria,^61,140,141^ such as *Lysinibacillus parviboronicapiens* whose ice-nucleating activity appears to be based on polyketides rather than proteins.^141^ Even among known bacterial *ina* genes, there are stark similarities but also some differences in genetic domains^142,143^ between the *inaZ*,^101^ *inaC*,^144^ *inaK*,^145^ *inaV*,^146^ and *inaQ*^147^ genes of *P. syringae*; *inaA* of *Erwinia ananas*^148^ and *Pantoea ananatis* (formerly *Erwinia uredovora*);^149^ *inaW* of *Pseudomonas fluorescens*;^150^ *inaU* of *Pantoea ananatis*;^151^ *inaX* of *Xanthomonas campestris* pv. *Translucens*;^152^ *inaPb* of *Pseudomonas borealis (P. borealis)*;^153^ and *inaE* (*iceE*) of *Pantoea aggolerans* (formerly *Erwinia herbicola*).^154^

The tools are not currently available for achieving systematic identification and quantification of biological and biogenic INPs in a format that can be employed broadly throughout the community. This particularly applies to field campaigns where sample volumes, sample throughput, and automation become important.

Microfluidic technology offers a means of revolutionizing INP analysis by enabling the use of powerful bioanalytical techniques developed and refined over three decades^155^ for a broad range of samples and analytes.^156–159^ These lab-on-a-chip devices typically comprise networks of micrometer-scale channels within which fluids can be controlled and manipulated, allowing for sample processing, treatment, and analysis. The ability to integrate actuation mechanisms and detection systems, together with the ability to perform rapid chemical reactions on small sample volumes, allows for automated, small footprint, portable devices that have been developed for point-of-care diagnostics in the field of clinical testing.^160–163^

Microfluidic technology has been applied throughout several areas of environmental analysis,^164–166^ including for continuous and automated monitoring in the field,^167–171^ for example, in water analysis.^172–176^ It has also been applied to various aspects of bioaerosol sampling and analysis, and the reader is directed to more general reviews on microfluidic sampling and analysis of bioaerosols, pathogens, and particulate matter.^177–182^ Microfluidics have further been utilized on unmanned aerial vehicles (UAVs), i.e., drones.^183,184^ UAVs have also shown great potential for atmospheric aerosol^185^ and INP analyses in recent years,^186–190^ alongside balloon-borne instrumentation.^191,192^

**FIG. 1.**
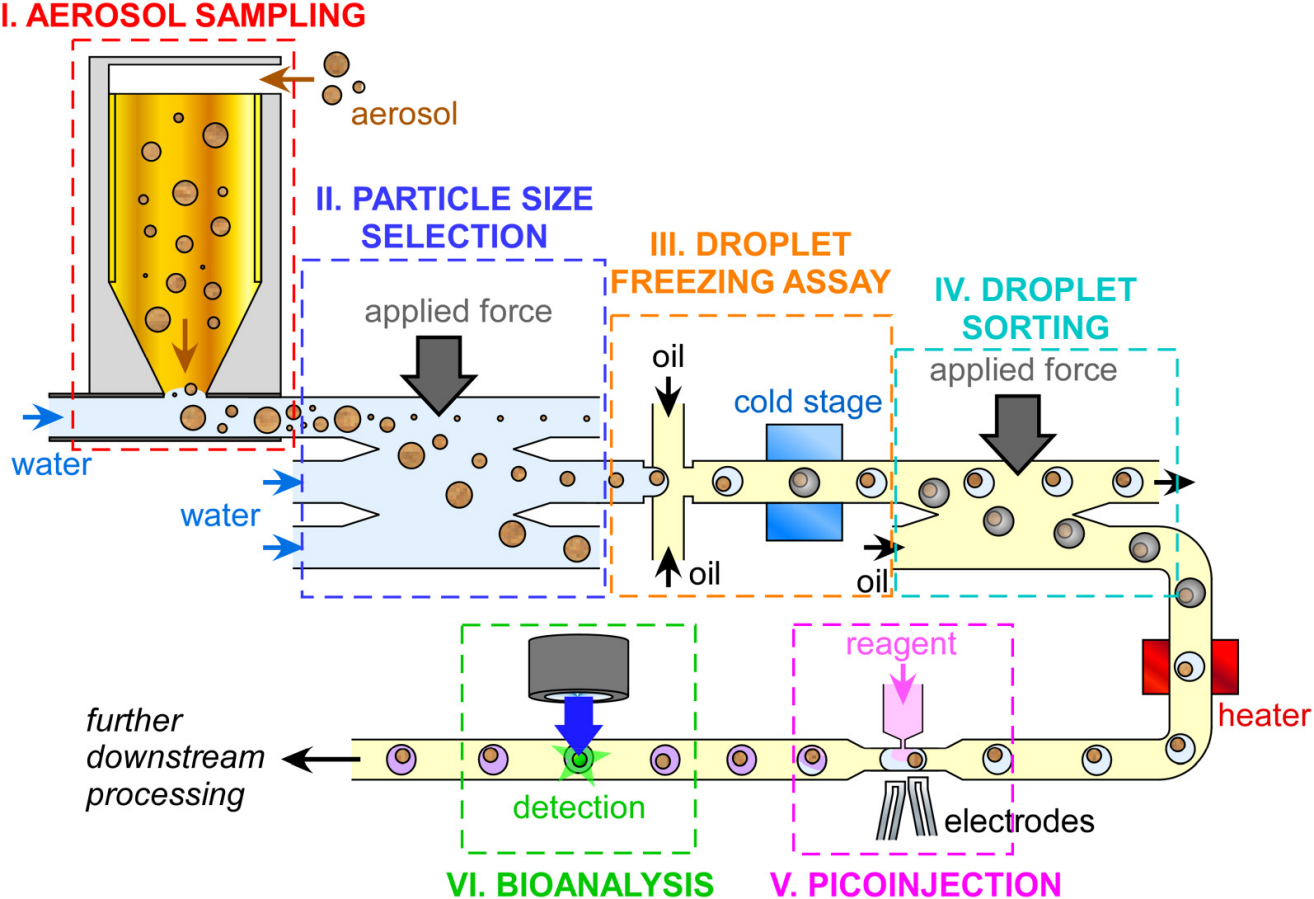
An idealized example of a sample-to-answer microfluidic platform for the sampling and analysis of biological ice-nucleating particles (INPs) incorporating all of the major processes, including (i) aerosol samplng, (ii) particle size separation and selection, (iii) droplet freezing assay (DFA) for INP quantification, (iv) separation of frozen and unfrozen droplets, (v) picoinjection of biochemical reagents into droplets, and (vi) bioanalytical identification and quantification of biological species via methods such as immunoassays or DNA analysis.

Here, we discuss the potential of microfluidic and lab-on-a-chip technology to revolutionize the monitoring of biological INPs in the atmosphere, focusing on the core aspects of (i) aerosol sampling, (ii) aerosol particle separation, (iii) determination of INP concentrations, (iv) separation of INP populations, (v) injection of chemicals for bioanalytical testing, and (vi) identification and quantification of biological INPs. With the integration of all of these steps into one apparatus, we can envisage an all-in-one, automated, sample-to-answer platform (see Fig. 1): a micro total analysis system (*μ*TAS)^156^ for the quantification and characterization of INPs.

We note that this article is not intended to be an extensive review of all of the relevant microfluidic literature pertaining to aerosol sampling and bioanalysis as such an endeavor would be overwhelming; rather, it will provide an overview of viable microfluidic strategies regarding each of the key analytical steps defined above. Where possible we also provide citations to review articles that provide a more detailed discussion of the theory, operation, and application of specific techniques. Hereafter, we cover the following topics associated with building a sample-to-answer microfluidic biological INP analysis platform (also see Fig. 1), followed by a discussion of the challenges and considerations surrounding the development of such a system:

I. Miniaturized bioaerosol sampling
II. Particle size separation
III. Microfluidic ice-nucleating particle analysis
IV. Microfluidic droplet sorting
V. Droplet picoinjection
VI. Microfluidic bioaerosol analysis

## II. MINIATURIZED BIOAEROSOL SAMPLING

The first crucial step in bioaerosol analysis is the sampling method, which must provide excellent collection efficiency over a wide range of particle diameters, and for which there are many different methodologies,^133,181,193,194^ all of which could be used to collect aerosols for offline transfer into microfluidic analysis systems. Microfluidic analysis must also compromise the inherent small volume analysis with possible low concentrations of analytes, which could result in non-detection of the target without performing a whole sample analysis. Nonetheless, a number of microfluidic strategies have been developed for efficient bioaerosol sampling, and these are covered more thoroughly in focused reviews.^179,180,182,195,196^ We also note that personal aerosol samplers, which have suffered similar drawbacks in the past, are now relatively low cost, small, and efficient^197,198^ and could be applied to microfluidic analyses in the future.

Here, we cover the common sampling strategies that are used throughout the INP community and provide an overview of microfluidic bioaerosol sampling techniques that could be applied to INP analysis.

### A. Traditional filter sampling

**FIG. 2.**
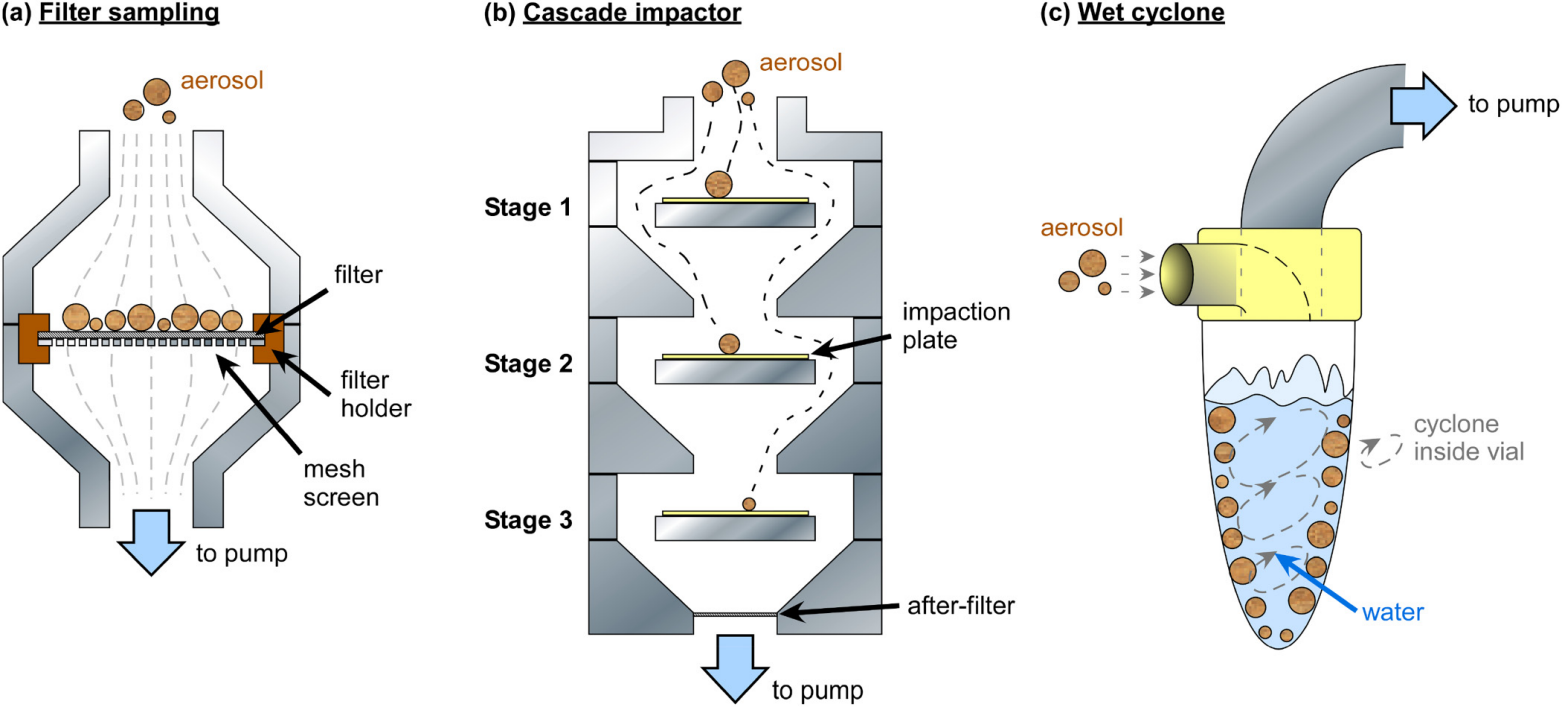
Traditional aerosol sampling techniques that have been employed for the microfluidic analysis of INPs. (a) Filter sampling, in which aerosols are pulled through a filter for the collection of particles, which are subsequently washed off the filter and into an aqueous suspension for analysis. Filter sampling is discussed further in Sec. II A. (b) A cascade impactor, in which aerosols pass through a series of nozzles and aerosols of differing size impact upon different collection plates. A three-stage impactor is shown. Plates (often small-pore filters) are then washed into aqueous suspension for analysis. Cascade impactors are discussed further in Sec. II C. (c) A wet cyclone sampler that pulls aerosols directly into water circulating within a vial, allowing a direct analysis of the aqueous suspension. Impingers are similar instruments in which air is bubbled into water, allowing transfer of the aerosols from the gas phase and into the aqueous phase (e.g., Greenburg–Smith bubble impingers). Wet cyclones are discussed further in Sec. II E.

Aerosol filter sampling has been employed for INP analysis since the 1960s^199,200^ and is now a staple of atmospheric INP analysis. Here, air is drawn through a filter onto which aerosols are deposited, typically via an inlet head that controls the size of the particles being collected [e.g., total suspended particulates (TSPs), or particulate matter smaller than 10, 2.5, or 1 *μ*m (PM_10_, PM_2.5_, PM_1_)], allowing recovery of the aerosol for offline analysis [Fig. 2(a)]. While many filter sampling instruments can be bulky, it is also possible to use small, lightweight setups for aerosol collection,^191,192^ which provides a powerful option for an integrated and miniaturized platform.

Sample filters collected for INP measurements would be immersed in a known volume of water and agitated by vortexing or shaking to release the collected aerosol particles into an aqueous suspension for offline analysis. This technique has been applied successfully in microfluidic droplet INP analysis, for example, by Tarn *et al.*^201,202^ in the droplet emulsion-based “Microfluidic pL-NIPI” (Picolitre Nucleation by Immersed Particle Instrument) instrument and the continuous flow “LOC-NIPI” (Lab-on-a-Chip Nucleation by Immersed Particle Instrument),^203^ and by Brubaker *et al.*^204^ and Jahl *et al.*^205^ in their microfluidic droplet array-based “store and create” platform.

However, due to ambient aerosol concentrations and sample volumes compared to the small volumes of a microfluidic droplet freezing device, the resultant INP spectra tend to cover the colder temperature regions, where higher concentrations of lower activity INPs are expected. This issue can be addressed by analyzing more droplets, i.e., more or all of the sampled volume, in order to detect the rarer but more highly active INPs, which is a strategy more suited to continuous flow microfluidic systems where the user can define the number of droplets to be analyzed.^203,206,207^

### B. Microfabricated filter sampling

**FIG. 3.**
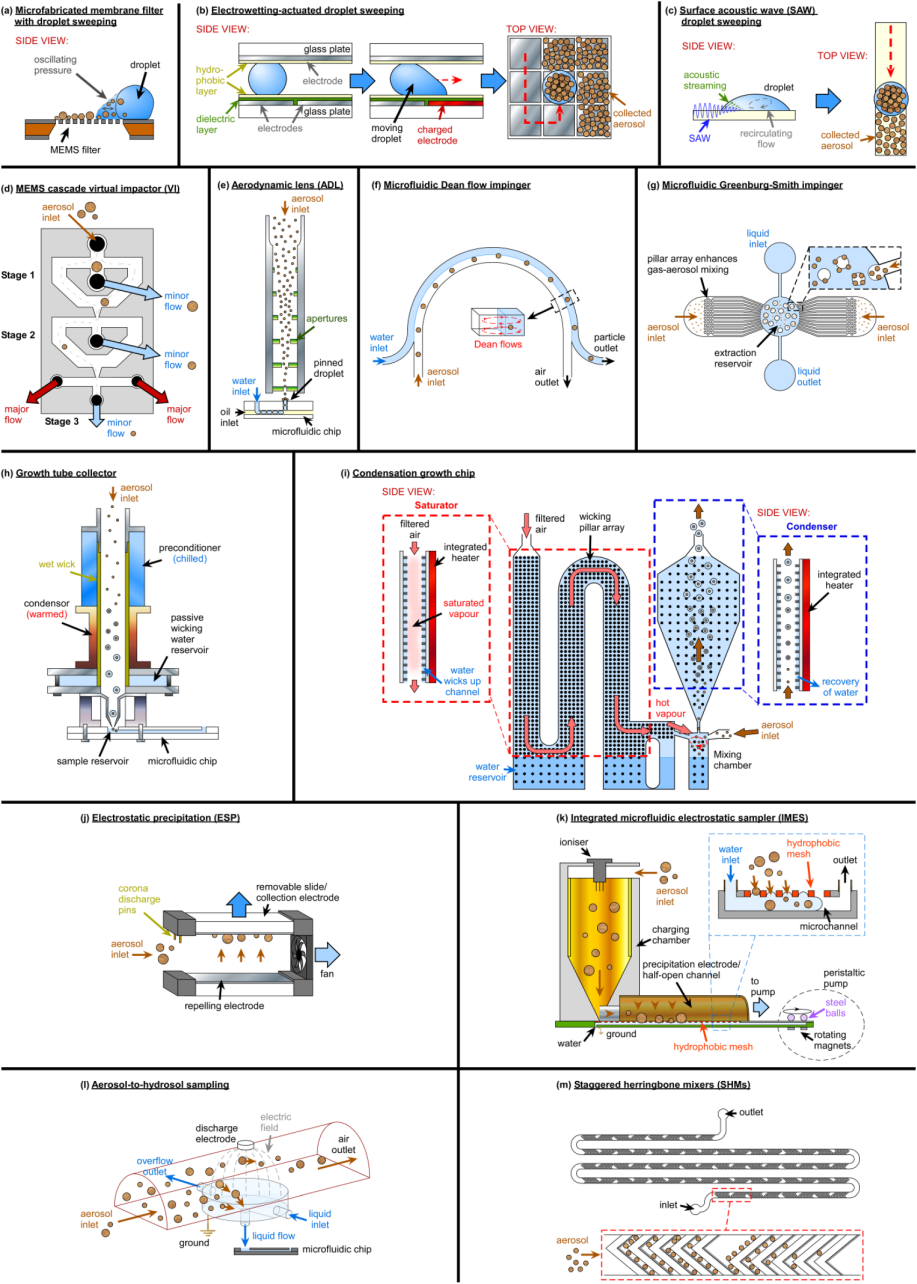
Various miniaturized aerosol sampling techniques that have been developed to collect aerosol particles directly into aqueous suspensions in microfluidic platforms. (a)–(c) Examples of the “droplet sweeping” technique for the collection of aerosols into a droplet, upon prior sampling of the aerosols onto a microfabricated filter or a flat substrate. (a) A microfabricated membrane filter, with a droplet actuated by oscillating pressure to drive the air–liquid interface over the region of captured droplets.^208^ (b) Principle of electrowetting-on-dielectric (EWOD), in which droplets can be moved across arrays of electrodes, to sweep up collected particles.^212^ Adapted and used with permission from Zhao and Cho, Lab Chip **6**, 137–144 (2006). Copyright 2006 Royal Society of Chemistry, permission conveyed through Copyright Clearance Center, Inc. (c) Surface acoustic waves (SAWs) applied via transducers to generate recirculating flows in a droplet to sweep up particles.^232^ Adapted and used with permission from Tan *et al.*, Lab Chip **7**, 618–625 (2007). Copyright 2007 Royal Society of Chemistry, permission conveyed through Copyright Clearance Center, Inc. (d) Microelectromechanical systems (MEMS)-based virtual impactors (VIs).^233^ Reprinted with permission from Kim *et al.*, Appl. Phys. Lett. **91**, 043512 (2007). Copyright 2007 AIP Publishing LLC. (e) An aerodynamic lens (ADL) that directs a narrow band of aerosol into a pinned droplet.^234^ Adapted and used with permission from Damit *et al.*, Aerosol Sci. Technol. **51**, 488–500 (2017), reprinted by permission of the publisher (Taylor & Francis Ltd, http://www.tandfonline.com). (f) A curved microfluidic impinger that employs Dean forces to continuously transfer aerosols into water.^235^ Reprinted (adapted) with permission from Choi *et al.*, ACS Sensors **2**, 513–521 (2017). Copyright 2017 American Chemical Society. (g) A microfluidic Greenburg–Smith impinger in which bubbles of aerosol are generated in liquid for their transfer into aqueous suspension.^236^ Adapted and used with permission from Mirzaee *et al.*, Lab Chip, **16**, 2254–2264 (2016). Copyright 2016 Royal Society of Chemistry, permission conveyed through Copyright Clearance Center, Inc. (h) A traditional condensation growth tube collector integrated with a microfluidic device.^237^ Reprinted (adapted) with permission from Noblitt *et al.*, Anal. Chem. **81**, 10029–10037 (2009). Copyright 2009 American Chemical Society. (i) A microfluidic condensational growth chip.^238^ Adapted and used with permission from Kwon *et al.*, Lab Chip **19**, 1471–1483 (2019). Copyright 2019 Royal Society of Chemistry, permission conveyed through Copyright Clearance Center, Inc. (j) An electrostatic precipitator (ESP) with a removable collection slide that can be subjected to droplet sweeping particle collection, e.g., via EWOD.^239^ (k) An integrated microfluidic electrostatic sampler (IMES).^240^ Adapted and used with permission from Ma *et al.*, J. Aerosol Sci. **95**, 84–94 (2016). Copyright 2016 Elsevier*.* (l) An aerosol-to-hydrosol sampler employing ESP.^241^ Adapted and used with permission from Park *et al.*, Anal. Chim. Acta **941**, 101–107 (2016). Copyright 2016 Elsevier. (m) Staggered herringbone mixer (SHM) micropatterned grooves in a microfluidic device that generate chaotic flows for the capture of aerosol in the grooves, allowing them to be washed into aqueous suspension.^242^ Reprinted (adapted) with permission from Jing *et al.*, Anal. Chem. **85**(10), 5255–5262 (2013). Copyright 2013 American Chemical Society.

Microelectromechanical system (MEMS) devices have been developed toward enabling the collection of aerosol onto microfabricated membrane filters [Fig. 3(a)], though have largely not been used in such a manner. Rather, methods to “sweep” collected particles from a microfabricated filter or a solid substrate and across an air–liquid interface into a droplet following the aerosol collection step have been explored [Figs. 3(b) and 3(c)].

Desai *et al.*^208,209^ developed a glass and polydimethylsiloxane (PDMS)-based “active filter membrane” to provide an air-to-liquid particle capture scheme. Here, particles would be collected onto a microfabricated membrane filter grid then, in principle, dielectrophoretic (DEP) forces would be applied via electrodes to drive the collected aerosol particles across an oscillating air–liquid interface to “sweep” the particles into a liquid droplet, thus generating an aqueous suspension. However, the filter sampling aspect was not tested, with the focus of the work being on the transfer of particles across the interface via an external pressure source (rather than DEP) [Fig. 3(a)]. Collection efficiencies were low, but the principle showed promise.

Zhao and Cho^210–212^ developed a silicon-based microfabricated filter that was integrated into an electrowetting-on-dielectric (EWOD) system.^213,214^ EWOD comprises an array of patterned electrodes on which single droplets can be manipulated and moved by applying electric fields [Fig. 3(b)]. Here, particles collected onto the filter could be “swept” into droplets as they were moved across the electrode array. Although the filter sampling aspect was not performed here, the method was found to provide high collection efficiency.

This “droplet sweeping” method^215^ may alleviate some of the issues with using traditional filter sampling with microfluidic INP analysis by entraining collected aerosol particles into a much smaller sampler volume, thus achieving a more concentrated suspension.

Liu *et al.*^216^ fabricated a microfluidic module that contained a semiporous PDMS filter membrane within it for the collection of bioaerosols and waterborne pathogens for DNA analysis. *Pseudomonas aeruginosa (P. aeruginosa)*, a known INP,^217^ was aerosolized and drawn through the filter via a vacuum pump, then the collected bacteria were lysed to release target DNA that was detected via on-chip loop-mediated isothermal amplification (LAMP).

### C. Cascade impactors

Impaction of aerosols is the method by which air is accelerated through an orifice and then *over and around* a plate or substrate (rather than *through* the substrate as in filter sampling), with particles larger than a critical size (determined by the orifice and speed of air-flow) having enough momentum that they impact on the plate while smaller particles continue to follow the air stream.^218,219^ A single stage impactor can be used to cutoff particle collection above a particular size threshold. By having a series of orifices that provide greater jet speeds, resulting in smaller and smaller critical particle sizes, and an impaction plate between each orifice, particles of different size classes can be collected onto the plates for size-segregated analysis [Fig. 2(b)]. This methodology has proven useful in INP measurement campaigns for determining the size-resolved ice-nucleating activity of ambient samples.^47,56,191,220–229^

Creamean *et al.*^47^ have employed a four-stage time-and-size-resolved Davis Rotating-drum Universal-size-cut Monitoring (DRUM) single-jet impactor^230^ to collect aerosols in four size ranges for INP analysis. The DRUM impactor collects size-resolved aerosols onto Vaseline-coated Mylar tape attached to rotating drums, allowing the sample to be deposited on the moving tape to enable time-resolved collection. Other impactors used in aerobiology include the Burkard or Hirst type spore trap, which uses a single impactor stage in addition to rotorods or rotating-arm traps used as impaction substrates.^194^

Reicher *et al.*^203,220,221^ employed a commercial micro-orifice uniform deposit impactor (MOUDI)^231^ cascade impactor to collect aerosols onto filters (used as impaction substrates), for size-segregated INP analysis using their “WISDOM” (WeIzmann Supercooled Droplets Observation on a Microarray) droplet array microfluidic platform.

A number of miniaturized and MEMS cascade impactors have been developed to aid in portability and cost-effectiveness.

Maldonado-Garcia *et al.*^243^ designed a two-stage MEMS impactor in which the impactor plates were MEMS resonant microbalances capable of measuring the mass concentration of collected materials. Kang *et al.*^244^ developed a three-stage microfabricated cascade impactor whose particle diameter cutoffs were found to be very similar to numerically predicted values.

Kwon *et al.*^245^ fabricated a miniaturized, 3D printed cascade impactor system that incorporated sensing electrodes into the impactor plates. As aerosol entered the system, a unipolar mini-discharger was used to electrically charge the particles, allowing their detection on the electrodes as the different size fractions were collected onto the impactor plates.

### D. Virtual impactors

Virtual impactors (VIs) operate in a similar manner to cascade impactors, but rather than having aerosols impacting onto plates they instead enter a “virtual space” of slow moving air provided by a minor flow, allowing for the collection of particles, while a major flow drives uncollected particles further through the system.^246^ Large virtual impactors can sample high volumes of air, e.g., Burkard high-throughput jet samplers, since there are fewer restrictions on air flow than when using filters.^247^ While multiple pumps are usually required in order to operate a virtual impactor, one for the major flow and one for the minor flows of each virtual impactor stage, Kim *et al.*^233,248^ micromachined a three-stage virtual impactor [Fig. 3(d)] that featured a flow rate distributor. This avoided the need for multiple pumps by having the microfabricated distributor control the flow to each part of the virtual impactor system, thus requiring only one pump to operate the entire three-stage impactor. The same group also developed a single-stage micro virtual impactor^249^ that could be adapted to include cutoff diameters down to 15 nm by the application of electric fields via integrated electrodes to accelerate the smaller particles.^250,251^ The single virtual impactor was further integrated with a micro corona discharger that charged the separated particles and measured their number concentration based on the electrical current carried by the particles.^252^

Zhao *et al.*^253–255^ 3D printed a single-stage miniaturized virtual impactor that incorporated a quartz crystal microbalance (QCM) to detect the mass loading of the collected aerosol, later replacing the QCM with a surface acoustic wave (SAW) sensor.^256^ Kim *et al.*^257^ demonstrated how, by integrating electrodes into a microfabricated single-stage virtual impactor, the particle size cutoff could be tuned from 35 to 70 nm by applying an electric field.

Further microfluidic virtual impactors have since been developed or proposed to enhance the separation and collection efficiency of airborne particles across multiple impactor stages.^258–264^

Liu *et al.*^265^ developed a miniaturized virtual impactor for PM_2.5_ separation combined with a thermophoretic precipitator, which uses the Soret effect to move particles in a temperature gradient toward the colder region,^266^ to collect the particles for measurement of the mass loading on a SAW sensor.

### E. Impingers and wet cyclone samplers

Impingers and wet cyclone samplers are forms of aerosol sampler that collect particles directly into a volume of water using a pump, eliminating the need to wash particles off collection substrates as in the above examples. Such devices have been employed for the collection of aerosol for INP analysis,^42,203,267–269^ often allowing for higher sampling rates (e.g., hundreds of liters per minute) than commonly used filter samplers (e.g., ∼10–33 l min^−1^). A Greenburg–Smith bubble impinger, for example, involves bubbling sample air into a vial of water, allowing for aerosols in the bubbles to enter an aqueous suspension. Wet cyclone samplers rely on an airstream being directed tangentially into a tapering conical tube to create a vortex (e.g., the Coriolis Micro from Bertin Instruments).^270^ The conical tube contains liquid that swirls in the airflow to wet the inside of the device, causing particles to be deposited on the wet walls of the tube by their inertia [Fig. 2(c)].

A Coriolis Micro was used by Tarn *et al.*^203^ to collect aerosol particles at 300 l min^−1^ for INP droplet freezing analysis in the continuous flow LOC-NIPI. The Coriolis Micro has also been applied to the collection of bioaerosols for the detection of *Escherichia coli (E. coli)* via microfluidic cytometry.^271^ However, the technique suffers similar drawbacks for microfluidics analysis of rare particle types (e.g., INPs) as filter sampling, with the sample being drawn into a relatively large volume of water (∼5 ml), though impingers can be used to concentrate samples by evaporation of the working fluid during collection.

Several microfluidic impingers have been developed that enable the sampling of aerosol particles directly into water within a microchannel. Damit^234^ employed a commercially available aerodynamic lens (ADL),^272^ which uses a series of orifices to focus aerosol particles within a specific size range into a narrow stream, to direct aerosol particles directly into a pinned droplet of water in a detection channel of a microfluidic device for the detection of *E. coli* [Fig. 3(e)].

Choi *et al.*^235^ developed a continuous flow impinger based on a curved inertial microfluidics device [Fig. 3(f)]; while most microfluidic systems work within a regime of low Reynolds numbers (Re ≪ 1), inertial microfluidic devices operate in the intermediate regime (Re ≈ 1–100) that yields unique flow and particle phenomena at, typically, high flow velocities (e.g., tens of meters per second).^273,274^ One such effect is the migration of particles to equilibrium positions within a microchannel as they flow,^275^ with the position depending on the particle size and shape.^276^ In curved channels, secondary flows are generated based on the relative inertia of the fluid at different points in the bend, inducing Dean vortices to generate new equilibrium positions.^277^ The device of Choi *et al.*^235^ utilized these forces within a stratified flow of air and water to transfer aerosol into suspension as they flowed around the curved channel, allowing for the sampling of *Staphylococcus epidermidis (S. epidermidis)* and its off-chip analysis by fluorescence microscopy. The Dean flow impinger was later updated to incorporate surface-enhanced Raman spectroscopy (SERS), with silver nanoparticles (AgNPs) pumped through the device to bind to the impinged aerosols, allowing their *in situ* detection as they passed through a Raman detection region.^278^ This optofluidic SERS platform was used for the sampling and identification of several bacteria (*S. epidermidis*, *Micrococcus luteus (M. luteus)*, *Enterococcus hirae (E. hirae)*, *Bacillus subtilis (B. subtilis)*, and *E. coli*), including the quantification and real-time monitoring of *S. epidermidis*.

Mirzaee *et al.*^236^ demonstrated a miniaturized version of a Greenburg–Smith bubble impinger [Fig. 3(g)]. Here, microfluidic channels were used to generate bubbles laden with aerosol into a central extraction reservoir and demonstrated high sampling efficiency.

Thus, there are several means by which atmospheric INPs could potentially be drawn directly into microfluidic devices and deposited directly into liquid for ice nucleation and biological analysis.

### F. Dry cyclone samplers

While dry cyclones have not been applied to INP sampling, to our knowledge, or for microfluidic purposes, adaptation of the methodology could enable both. Dry cyclone samplers were first developed in the 1950s and rely on a vortex of air being created inside a dry cylinder that transitions to a tapering conical tube, ending with a removable sample tube. Particles separate from the air flow into the dry collection tube as the air suddenly changes direction from the tightening vortex to flow vertically upwards through the center of the vortex.^279^ While collection efficiencies can be low for small particles, high sampling rates can be achieved, allowing for the collection and analysis of, for example, fungal spores by qPCR analysis.^280^ Dry cyclones typically result in a collection of dust, pollens, and spores in a dry tube format, which is convenient for a wide range of downstream diagnostic and analytical applications and would be highly amenable to the droplet sweeping aerosol-to-water collection method.

### G. Condensation growth tube collectors

A growth tube aerosol collector-based system was developed by Noblitt *et al.*^237^ to sample aerosol directly into a microfluidic device for electrophoretic separation and detection of inorganic ions. The system employed the growth tube from a conventional water-based condensation particle counter (WCPC), which sampled air through a wet-walled tube comprising cool and warm regions, such that aerosols passing through the tube would experience condensational growth to the supermicrometer size range [Fig. 3(h)]. The aerosols were then deposited into a buffer-filled microfluidic reservoir for analysis. The system collected aerosols at 1 l min^−1^ into 30 *μ*l of buffer and ran continuously for 28 h taking measurements. Given its basis on instrumentation found in commercial CPCs, it may be possible to adapt such technology for continuous sampling of INPs into microfluidic devices.

Kwon *et al.*^238^ developed a MEMS-based condensation growth chip to grow nanoparticles (5–80 nm) to micrometer sized droplets. The chip comprised micropillar arrays throughout that allowed water to be wicked through the device from a reservoir, forming water-lined walls between which aerosols could pass [Fig. 3(i)]. By integrating MEMS heaters and temperature sensors, regions of vapor saturation, aerosol introduction, and condensational growth were incorporated into the planar device, allowing downstream detection via a miniaturized optical particle counter (OPC).

### H. Electrostatic precipitation

Electrostatic precipitation (ESP) is a sampling technique in which aerosols drawn through an inlet are charged by corona discharge needles and then deposited onto a hydrophobic surface (the ground electrode), allowing for the collection and analysis of the particles. Such devices have been employed for INP analysis on conventional measurement platforms,^189,281^ and the technique is amenable to miniaturization for microfluidic applications.^239^

Sandstrom *et al.*^282^ developed a microfluidic air–liquid interface by fabricating a liquid ground electrode device, such that the particles charged by the corona needle were directly deposited into an electrolyte solution through a microfabricated silicon diaphragm. Pardon *et al.*^283^ further developed this platform to incorporate the ESP concept into a miniaturized package for point-of-care (POC) sampling of aerosols that could be integrated with a silicon diaphragm-based microfluidic device.

Foat *et al.*^239^ developed an EWOD platform integrated with a miniaturized ESP sampler such that aerosol was precipitated onto the electrode array substrate. The substrate comprised a plate that could be removed from the sampler and inserted into an EWOD device [Fig. 3(j)], allowing EWOD actuation of droplets to “sweep up” the collected particles^215^ in a similar manner to the microfilter-EWOD device of Zhao *et al.* [Fig. 3(b)].^210–212^

Ma *et al.*^240^ utilized a half-open microchannel to collect precipitated aerosol through a mesh, allowing their transport to a collection reservoir in their “integrated microfluidic electrostatic sampler” (IMES) platform [Fig. 3(k)]. Shen *et al*.^284^ combined an ESP system to deposit aerosol directly into liquid in a charged reservoir, with a peristaltic pump used to transfer the liquid continuously to a silicon nanowire field effect transistor for real-time airborne influenza monitoring.

Park *et al.*^241^ used ESP-based aerosol-to-hydrosol (ATH) sampling to collect test bacteria (*S. epidermidis*) into flowing liquid for on-chip analysis via an adenosine triphosphate (ATP) bioluminescence assay monitored using a photodetector [Fig. 3(l)]. Kim *et al.*^285^ later introduced a hydrosol-to-hydrosol (HTH) bacterial enrichment step in which magnetic particles were used to capture and concentrate the sampled bacteria and improve detection sensitivity via fluorescence microscopy, electrical detection, and qPCR DNA analysis.

### I. Surface acoustic waves

It has been demonstrated above that EWOD can be used to transfer particles collected via microfabricated filters^210–212^ or ESP substrates^215,239^ into droplets. SAWs are traveling waves that can be generated on piezoelectric substrates with integrated transducers and allow the movement of droplets using acoustic forces in a manner somewhat analogous to the electrically induced movement of EWOD [Fig. 3(c)]. Tan *et al.*^232^ demonstrated this capability by using SAWs to “sweep up” bioaerosols deposited on a substrate into a droplet and further showed that acoustically induced streaming and recirculation inside the droplets enhanced particle collection and efficiency. The device was not, however, integrated with an aerosol collection system, instead focusing on the post-sampling collection of particles into water, but demonstrated the potential of SAW for droplet sweeping of aerosols collected onto microfabricated filters or other substrates.

### J. Staggered herringbone mixers

Staggered herringbone mixers (SHMs) are periodic but alternating microfluidic channel structures, typically grooves, that induce chaotic mixing,^286^ traditionally to rapidly mix solutions for chemical reactions since the laminar flow nature of microfluidic flow usually limits mixing to slow diffusional processes. However, by drawing air through SHM devices under vacuum, it has been demonstrated that airborne bacteria can be captured and concentrated in the grooves [Fig. 3(m)],^242^ then eluted for on-chip immunoassay analysis^287^ or on-chip DNA analysis by LAMP analysis of the ice-nucleating bacteria, *P. aeruginosa*.^288,289^

Bian *et al.*^290^ demonstrated the enhancement of SHM-based aerosol collection by adding spiral microchannel-induced inertial forces and sawtooth wave-shaped walls to better accommodate aerosol particles for off-chip analysis by liquid chromatography–mass spectrometry (LC–MS) and colony forming unit (CFU) assays.

## III. PARTICLE SIZE SEPARATION

**FIG. 4.**
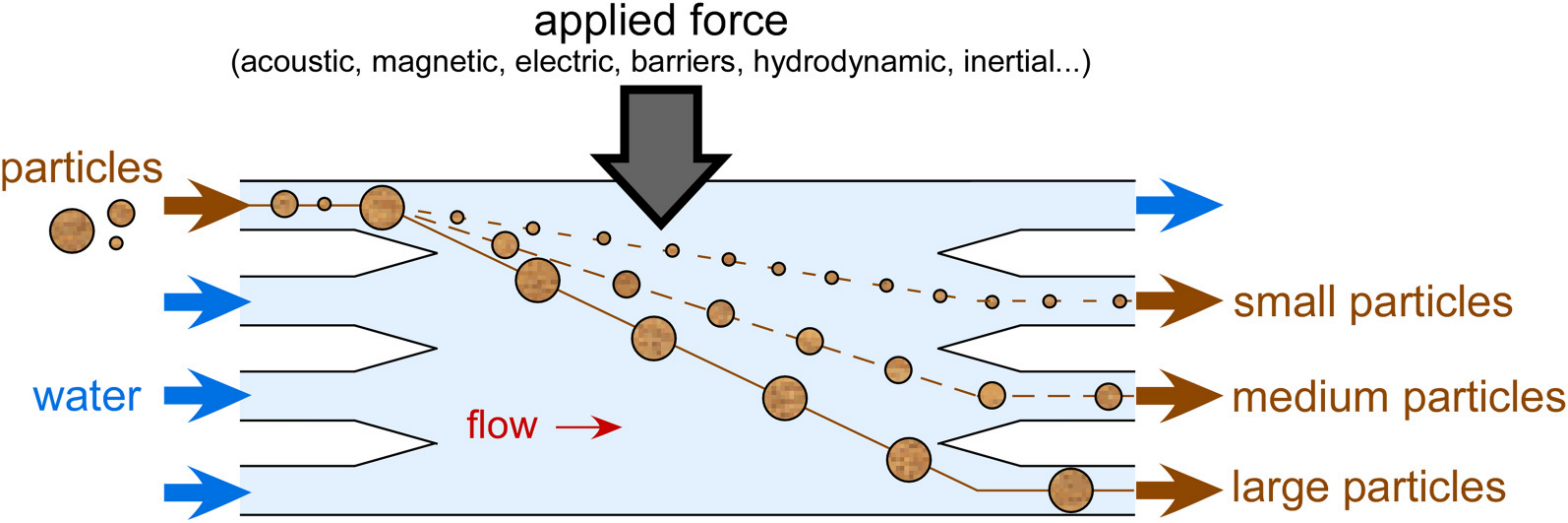
Principle of on-chip continuous flow size-based particle separations. Typically, a lateral force is applied to particles flowing across a microfluidic chamber, with different sized particles interacting with the force to differing extents. This allows some particles to migrate further in the lateral direction than others, enabling their collection via different outlet channels. Forces that can be utilized to induce the lateral flow include acoustic, magnetic, electric (e.g., dielectrophoretic), and hydrodynamic, or the use of pillars and barriers in the flow stream.

Once aerosol has been collected and, typically, processed into a volume of liquid to prepare a particle suspension, it may be desirable to separate particle populations based on size for INP analysis. This may be to perform on-chip size-resolved INP assays as per Reicher *et al.*^220,221^ or to filter out larger bioaerosols (e.g., bacteria, pollen grains) in order to analyze only the INM content. Other mechanisms for separation could also be based on morphology, charge, hydrophobicity, or relative deformability or compressibility, for example, to separate solid inorganic materials such as mineral dust from more pliable bioaerosols. Particle separations in continuous flow have proven to be a huge strength of microfluidics, often taking advantage of the laminar flow streams of fluid in such devices to transfer analytes of interest from one stream to another via a lateral force or barriers (Fig. 4), often for biomedical purposes.

As such, the scope for viable particle size separation techniques is far too large to cover in detail here, and the reader is directed to other dedicated reviews on the topic.^291–294^ Suffice to say, a range of active^295^ and passive^296^ techniques can be readily applied to microfluidic separations, including inertial forces,^297,298^ pillars and barriers,^299^ magnetism,^300^ acoustic forces,^301^ optical forces,^302^ and dielectrophoretic (DEP) forces.^303^ Here, we describe only specific examples of aerosol separations achieved in microfluidics, and note that microfluidic and MEMS devices for the separation and detection of aerosols have also been reviewed by Poenar.^304^

*Cascade impactors*, described in Sec. II C, and *virtual impactors (VIs)*, described in Sec. II D, also provide particle size segregation as part of their collection process and are not described again here.

### A. Inertial microfluidics

As described earlier, inertial microfluidics can be used to manipulate particles at high flow rates such that they migrate to equilibrium positions [see Fig. 3(f)],^273,274^ and by including multiple outlet channels within the microfluidic design it is, therefore, possible to separate and collect particles that have been stratified.

Schaap *et al.*^305,306^ demonstrated the use of both straight and curved inertial microfluidic devices to perform size-based separations of aerosol particles across sheath air streams, which compared well with simulations, while Hong *et al.*^307^ demonstrated multi-stage separation via consecutive curved channels. Various numerical analyses and simulations have also been performed of the inertial migration of aerosol particles in microfluidic channels^305,306,308^ and capillaries.^309–311^

Inertial microfluidics offers a rapid and passive means of achieving separations, although the high flow rates required to generate the requisite forces may not always be made easily compatible with upstream or downstream processes within an integrated platform.

### B. Deterministic lateral displacement

Deterministic lateral displacement (DLD) is a technique that employs a regular array of micropillars to separate particles in continuous flow.^299,312,313^ Each row of the array is laterally shifted, generating distinct flow patterns between each pillar. As particles migrate with the flow stream, particles above a critical size are “bumped” laterally by the pillars while smaller particles continue in the direction of the flow, thus achieving separation. DLD is a powerful tool that has been applied to the separation of a range of biological, though the design of the pillar geometry for a critical size must be carefully determined, while complex biological matrices can foul and clog such platforms.

Yin *et al.*^314^ fabricated a DLD platform comprising I-shaped pillars for the separation of PM_2.5_ aerosols followed by electrochemical detection with commercial screen-printed electrodes (SPEs). The separation of 1 and 10 *μ*m polystyrene particles was achieved with nearly 100% efficiency, though particles between these sizes or bioaerosols were not tested.

### C. Electrical mobility analyzer

While not strictly microfluidic, Kwon *et al.*^315^ reported a micromachined nano-electrical mobility analyzer (NEMA) for separating and classifying nanoscale (<100 nm) aerosol based on aerodynamic and electrostatic forces. The NEMA was fabricated from silicon and featured integrated electrodes that applied an electric field across a microchannel, such that particles migrated laterally toward a ground electrode as they flowed through the channel. Larger particles with more inertia would be less affected by the electric field, thus exiting via a bypass outlet, while small particles would migrate toward and be captured at the ground electrode. By applying specific voltages, target particles could be manipulated into a collection outlet based on their size and charge for further processing. In many ways, the NEMA operated in a similar manner to the micro free-flow electrophoresis (FFE) continuous flow separation technique,^316^ but while the latter employs an electrolyte solution to separate target analytes the NEMA operated in air to process aerosol.

## IV. MICROFLUIDIC ICE-NUCLEATING PARTICLE ANALYSIS

A key measurement in atmospheric INP analysis is the determination of the concentration of INPs collected in ambient samples. There are several techniques and instruments available for achieving this,^268,317–319^ but arguably the workhorse of INP analysis is the droplet freezing assay (DFA) or droplet freezing technique (DFT) developed by Vali in 1971.^320^ In the standard technique, an array of aqueous droplets containing sample is deposited (typically via pipetting) onto a hydrophobic substrate, then cooled to around −40 °C at a constant rate (usually 1 °C min^−1^). The temperatures at which the droplets freeze are recorded, and these can be used to calculate the number of INPs within the volume of sampled air collected. This allows the determination of an “INP spectrum,” a plot of INP concentration vs the temperature at which those INPs are active, i.e., the temperature at which they trigger freezing. INPs active at warmer temperatures (closer to 0 °C) tend to be rarer, while those active at colder temperatures tend to have much higher concentrations. DFAs can be used to determine INP concentrations from environmental samples or from lab-prepared samples and standards. Knowledge of key parameters such as sample mass or surface area allows normalization of the DFA data to allow comparison of ice-nucleating activities in terms of the ice-active mass density, *n*_m_(*T*), or ice-active surface site density, *n*_s_(*T*). A number of variations and improvements have been made to DFAs over the years via the development of new instrumentation (overviews of modern instrumentation can be found in the literature,^317,321–323^ most recently by Miller *et al.*),^268^ but the core principles of DFAs remain the same.

Purified water can be cooled to around −35 to −40 °C before freezing homogeneously,^322,324,325^ and so in principle this should define the “background” or “baseline” of the DFA. However, DFAs typically employ droplets in the microliter range, and droplets of purified water droplets tend to freeze below ∼−20 to −25 °C, thus restricting INP measurements to warmer temperatures than homogeneous freezing.^320,326,327^ This can be caused by a number of factors, including an increased chance of impurities in such relatively large droplet volumes or interferences from the hydrophobic substrates employed,^328^ although with great care and preparation it is possible to reduce these effects.^329^

However, DFAs that employ picoliter droplets can readily achieve homogeneous freezing of water; reducing the size of the droplets means less chance of impurities on a droplet-by-droplet basis, and the droplets are typically immersed in an immiscible oil that eliminates interferences and impurities from solid substrates. In the past, such droplets have been generated via nebulization^21,330–332^ or by emulsification with a vortex mixer,^327,333^ but these produce very polydisperse droplet populations and the former method, in particular, can be non-trivial.

**FIG. 5.**
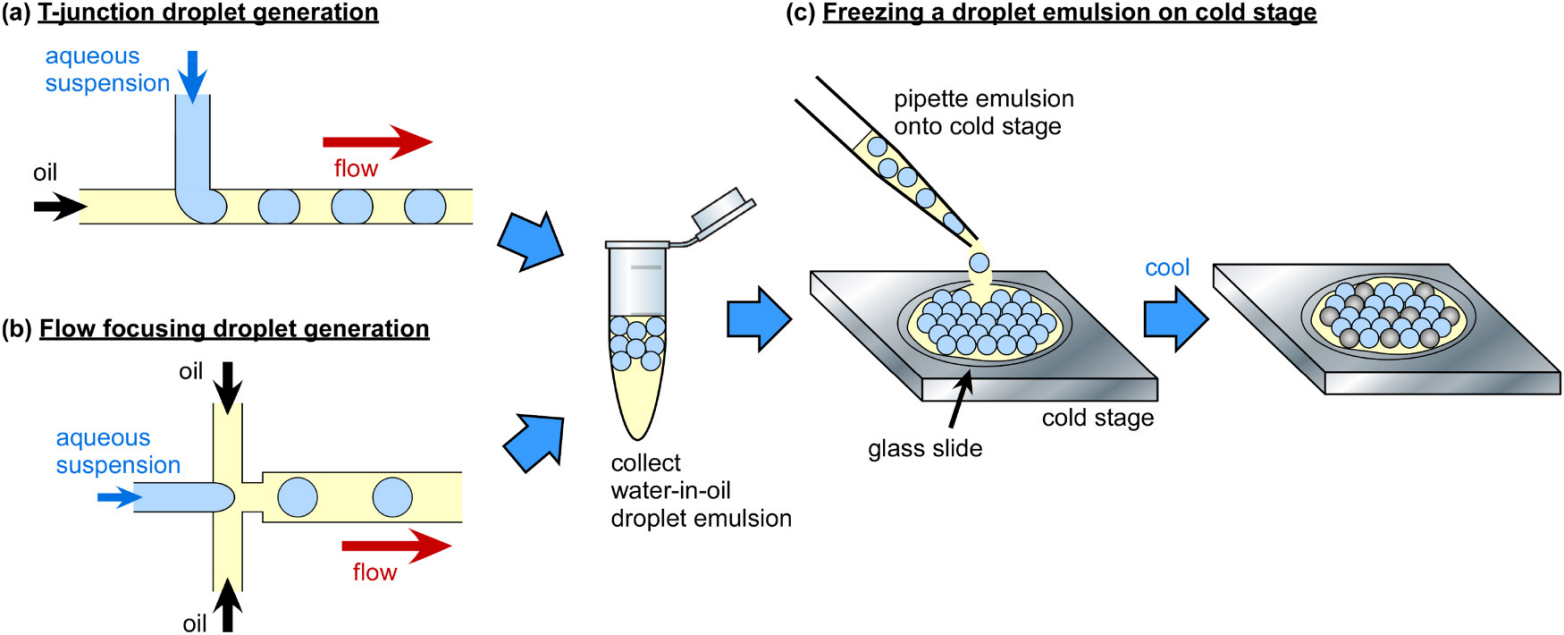
The use of microfluidically generated droplet emulsions for droplet freezing assays (DFAs). Water-in-oil droplets are typically generated in a (a) T-junction or (b) flow focusing channel configuration and collected off-chip in a vial. (c) The droplet emulsion can then be pipetted onto a glass slide on a cold stage and cooled until all of the droplets have frozen.^367^ The temperatures at which the droplets freeze reveal information on the concentration and activity (e.g., ice active site density per mass or surface area) of the INPs. A transparent lid is usally placed atop the droplet suspension during freezing (not shown for clarity) to prevent evaporation.

Microfluidics offers the capability to generate monodisperse water-in-oil droplets of controlled size easily and with high throughput.^334–336^ By pumping a liquid through a side channel (a T-junction)^337^ [Fig. 5(a)] or a nozzle (for flow focusing)^338^ [Fig. 5(b)], i.e., the discrete phase, into another flowing but immiscible liquid, i.e., the continuous phase, with a surfactant usually added to the immiscible phase to aid droplet stability, droplets of the discrete phase are “pinched off” due to the viscous forces. Droplet production rates of tens to thousands per second can be achieved relatively easily, while it is possible to produce >1 M per second.^339^ The integration of sensors and actuators into microfluidic platforms enables the manipulation, splitting, merging, trapping, separation, and counting of droplets for fields ranging from biochemical analysis to microparticle production via their use as reaction compartments.

The use of droplet microfluidics for ice nucleation builds on the back of centuries old discoveries.^340^ While Daniel Gabriel Fahrenheit was the first to describe the supercooling of rainwater in glass vials in 1724 (having observed the effect in 1721),^341^ Swiss physicist Albert Mousson discovered in 1858^342–344^ that water droplets smaller than 500 *μ*m (<65 nl) could be supercooled on hydrophobic surfaces. UK geologist and metallurgist Henry Clifton Sorby was the first to discuss, in 1859,^345^ the supercooling of water in capillary tubes, following a series of experiments in capillaries of 85, 36, and 25 *μ*m diameter, some of which were performed with the prominent Irish physicist, John Tyndall. Sorby noted, however, that “Dr. Percy,” likely in reference to UK metallurgist John Percy, had also observed the supercooling of water in capillary tubes. Dufour^346–348^ found in 1861 that water droplets in oil and chloroform emulsions could be easily supercooled to as cold as −20 °C. Modern day microfluidic ice nucleation, thus, continues the legacies of these findings via the supercooling of small water droplets in emulsions within microchannels.

The advantages of droplet microfluidics have seen an explosion of their use in recent years in the ice nucleation community for DFAs since the first demonstration of microfluidic freezing in 2007.^349^ The monodisperse picoliter droplets enable the homogeneous freezing regime to be accessed, thereby allowing INP spectra to be produced in the ∼−20 to −35 °C region that standard microliter DFAs cannot typically access.^322,328^ Furthermore, the acquisition of high quality droplet freezing data comprising hundreds or thousands of uniformly sized droplets allows for improved statistics compared to standard techniques with limited (e.g., <50) droplet numbers. For example, it enables the use of the differential nucleus spectrum, rather than the commonly used cumulative spectrum, both derived from the number of frozen droplets vs unfrozen droplets at a given temperature, for quantitative comparisons of ice-nucleating ability and the activation of ice-nucleating sites at specific temperatures. Differential spectra require the data to be binned into temperature intervals, hence experiments employing low droplet numbers suffer from a loss of fidelity upon binning. An in-depth discussion of the differential nucleus concentration is provided by Vali,^350^ who employed microfluidic DFA data from Polen *et al.*^328^ (using a microfluidic droplet array device discussed and validated by Brubaker *et al.*,^204^ described below) to illustrate its application and benefits. Fahy *et al.*^351^ recently demonstrated a method, based on empirical bootstrapping, for interpolating DFA data and deriving differential spectra with high confidence bands for quantitative comparisons, again taking advantage of the large droplet freezing datasets achievable with microfluidic instruments.

Several microfluidic DFAs have been developed and were recently reviewed by Tarn *et al.*^322^ in terms of their application to homogeneous freezing studies, and in a section in a review on microfluidic phase transfer studies by Roy *et al.*,^352^ but all also could or have been applied to the analysis of atmospheric INPs. We briefly describe the main strategies here, and an overview of microfluidic DFAs is provided in Table II in the Appendix. Most microfluidic DFAs employ water-in-oil emulsions, and a summary of suitable oils and surfactants for such emulsions and the lowest cooling temperatures achievable or tested are provided by Hauptmann *et al.*^353^ A number of reviews are also available that discuss various methods of microfluidic temperature control^354–356^ and measurement.^354,357^ Outside of ice nucleation, the study of the nucleation and crystallization processes in droplet microfluidics and their applications^358–363^ has been performed for a number of species, including proteins,^364^ acids,^365^ and inorganic crystallization,^366^ thanks to the high monodispersity achievable, the ability to control the microenvironment, and the high throughput that enables improved statistics.

**TABLE II.** Microfluidic droplet freezing assays (DFAs), their operating parameters, and samples that have been analyzed. Note that some instruments have been used for applications outside of DFAs, and these are indicated in italics. Where purified water has been used in multiple publications for background measurements, only the initial publication is provided unless a dedicated study on water was also undertaken. The number of droplets analyzed does not account for the theoretical number of droplets that could be analyzed, e.g., an instrument may hold 1000 droplets but if only 100 were observed under a microscope then the value of 100 is provided here.

Publication and technique	DFA type	Chip material	Droplet generation method	Droplet size (*μ*m)	Droplet volume (pl)	Droplets analyzed per DFA	Oil	Surfactant	Temperature uncertainty (°C)	Cooling rate (°C min^−1^)	Cold stage	Experiments/samples
**Droplet emulsions**												
Riechers *et al.* 2013^367^	Droplet emulsion	PDMS on silicon	T-junction	53 ± 6 to 96 ± 11	78 ± 30 to 463 ± 178	>1000	Methyl cyclohexane	2% w/w Span 80	±0.3	1	Linkam MDBCS196 cryostage or TA-Instruments DSC-Q100 differential scanning calorimeter	Homogeneous freezing of purified water^367^
Lignel *et al.* 2014^368^	Droplet emulsion	PDMS on glass	Flow focusing	60 to 80	113 to 268	>1000	Paraffin oil	1.8% w/w Span 80	N/A	0.5	Setaram Instrumentation microDSC7 evo differential scanning calorimeter	Purified water^368^
Weng *et al.* 2016^369^	Droplet emulsion	PDMS on glass	Flow focusing	35 ± 2	22 ± 5	200	3M^™^ Novec^™^ 7500 (HFE-7500) fluorocarbon	1.5% w/w Pico-Surf^™^ 1 (Sphere Fluidics)	N/A	1	Linkam FDCS196 cryostage	Purified water^369^
												Heavy water (D_2_O)^369^
												Snomax®^369^
												Ethylene glycol^369^
												Propylene glycol (PEG)^369^
												Trehalose^369^
Tarn *et al*. 2018;^201^ “Microfluidic pL-NIPI”	Droplet emulsion	PDMS on glass	Flow focusing	94 ± 3	435 ± 43	250–500	3M^™^ Novec^™^ 7500 (HFE-7500) fluorocarbon	2% w/w Pico-Surf^™^ 1 (Sphere Fluidics)	±0.5	1	TEC (aq. PPG cooled)	Purified water^201^
												Homogeneous freezing of water^201^
												Tap water^201^
												Snomax®^201^
												*B. pendula* (silver birch) pollen washing water^201^
												*F. avenaceum* fungi washing water^201^
												K-feldspar microcline BCS-376^201^
												UK rural (agricultural) aerosols^201^
												UK bonfire aerosols^201,202^
												Arctic sea surface microlayers (SMLs)^372^
												*S. marinoi* phytoplankton^372^
**Microfluidic droplet arrays**												
Edd *et al*. 2009;^373^ “Dropspots”	Microfluidic droplet array (based on Dropspots)^374^	PDMS on glass	Flow focusing	37 ± 2	26 ± 5	∼100	3M^™^ Fluorinert^™^ FC-40 fluorocarbon	PFPE-PEG block copolymer	N/A	0.6	Linkam FDCS196 cryostage	Purified water^373^
												Glycerol^373^
Sgro and Chiu 2010^381^	Microfluidic droplet array	PDMS on glass	Flow focusing	∼100	N/A	10s	Silicone oil (AR 20)	0.05% w/v Span 80	N/A	N/A	Helium gas cooled using LN_2_ and flowed through a chamber	Purified water^381^
												*Immiscible phase medium exchange* ^381^
Weng *et al.* 2018^382^	Microfluidic droplet array (employing droplets produced in Weng 2016 device)^369^	PDMS on glass	Flow focusing (from the Weng 2016 device)^369^	35 ± 2	22 ± 5	1500	3M^™^ Novec^™^ 7500 (HFE-7500) fluorocarbon	N/A (droplets stabilized in 1.5% w/w Pico-Surf^™^ 1 prior to introduction as an emulsion)^369^	N/A	1	Linkam FDCS196 cryostage	Purified water^382^
												Poly(vinyl alcohol) (PVA)^382^
												Poly(vinylpyrrolidone) (PVP)^382^
												Polyethylene glycol (PEG)^382^
Reicher *et al*. 2018;^220^ “WISDOM”	Microfluidic droplet array (based on Dropspots)^374^	PDMS on glass	Flow focusing	39 ± 3 or 96 ± 6	31 ± 8 or 463 ± 87	550 (for 39 *μ*m droplets) or 120 (for 96 *μ*m droplets)	Mineral oil	2% w/w Span 80	±0.3	1	Linkam THMS600 cryostage	Purified water^220^
												Homogeneous freezing of water^220^
												K-feldspar microcline BCS-376^220,317^
												NX illite^220,598^
												Arizona test dust (ATD)^220^
												Snomax®^317^
												Glucose^220^
												NaCl^220^
												Ammonium sulphate^220^
												Size-resolved eastern Mediterranean aerosol (dust storms)^203,220,221^
												*F. cylindrus* diatoms^32^
												Argentinian soil dust^317^
												Tunisian soil dust^317^
												Microcrystalline cellulose (MCC)^323^
												Nanocrystalline cellulose (NCC)^323^
												Poly(vinyl alcohol) (PVA)^380^
												Birch pollen washing water^380^
												*E. coli* (ArcticExpress strain)^379^
												Phosphate buffered saline (PBS)^375,379^
												Miller's LB broth medium^375,379^
												Bacterial ice-nucleating proteins (from *P. syrinage* and *P. borealis*)^375–377,379^
												Ice-binding proteins (from snow fleas)^378^
												Stochasticity and time dependence^598^
												Antifreeze proteins (type-III AFP, *Tm*AFP)^599^
												Secondary ice production^600^
Brubaker *et al*. 2020;^204^ “Store and create”	Microfluidic droplet array (based on store and create)^387^	PDMS	Store and create	300 *μ*m Ø × 95 *μ*m deep, or 450 *μ*m Ø × 95 *μ*m (pancake-shaped)	6 nl (for 300 *μ*m Ø) or 14 nl (for 450 *μ*m Ø)	40 (for 450 *μ*m Ø) or 720 (for 300 *μ*m Ø)	Squalene oil	None	±0.2	1	TEC cooled via a TECA AHP-1200CAS air chiller	Purified water^204^
												Interferences in purified water^328^
												NX illite^204^
												Snomax®^204^
												Biomass burning aerosol (BBA)^204^
												Aged BBA^205^
Tarn *et al*. 2021;^383^ “Store and create”	Microfluidic droplet array (based on store and create)^387^ on mineral thin section (based on Holden 2019)^394^	PDMS on mineral thin section	Store and create	∼110	∼700	100s	None (dry air or nitrogen gas)	None	±0.4	1	TEC (aq. PPG cooled)	K-feldspar mineral thin section^383^
Roy *et al.* 2021:^388^ “Store and create”	Microfluidic droplet array (based on store and create)^387^	PDMS on glass	Store and create	450 *μ*m Ø × 150 *μ*m deep (pancake-shaped)	21 nl	185	Silicone oil	None	±0.69	0.5	Linkam LTS420 cryostage	Purified water^388^
												Bulk seawater^388^
												Sea surface microlayers (SMLs)^388^
												Heat treated SMLs^388^
												Peroxide treated SMLs^388^
												Snomax®^389^
												Snomax® treated with cationic salts^389^
												Montmorillonite clay^390^
												Effects of pH, salinity, repeat freezing, and efflorescence–deliquescence (E–D) cycling on montmorillonite clay^390^
												Sodium chloride (freezing point depression)^390^
												*Crystallization and liquid–liquid phase transitions*^352^ *including in aerosols and SMLs*^388,391–393^
Eickhoff *et al*. 2023;^380^ “nanoBINARY”	Microfluidic droplet array (based on Dropspots^374^ and WISDOM^220^)	PDMS on glass	Flow focusing	96 ± 4	463 ± 58	70	3M^™^ Novec^™^ 7500 (HFE-7500) fluorocarbon	2% w/w PFPE-Tris	±0.3	1	Linkam BCS 196 cryostage	Purified water^380^
												Birch pollen washing water^380^
												Poly(vinyl alcohol) (PVA)^380^
												Ethanol^380^
												Propan-2-ol^380^
												1,3-Butanediol^380^
Printed droplet arrays												
Peckhaus *et al*. 2016;^384^ GeSiM droplet generator	Printed droplet array	Glass on silicon on piezoelectric transducer	Piezoelectric actuation	107 ± 14 (spherical cap)	215 ± 70	160–1500	Silicone oil (Rhodorsil 47 V 1000)	None	±0.1	1, 5, 10	Linkam MDBCS196 cryostage	Purified water^384^
												K-feldspar microcline FS02^a^ (BCS-376)^384^
												K-feldspar FS01^384^
												K-feldspar FS04^384^
												Na/Ca-feldspar FS05^384^
												Aluminum oxide (α-alumina)^395^
Kiselev *et al*. 2021;^396^ GeSiM droplet generator for mineral grain mounts and thin sections	Printed droplet array on mineral thin sections (based on Peckhaus 2016)^384^	Glass on silicon on piezoelectric transducer	Piezoelectric actuation	N/A	0.4 nl (grain mount); 1.4 nl (thin section)	380–540 (grain mount); 50–340 (thin section)	None	None	N/A	3	Linkam MDBCS196 cryostage	Treated Volkesfeld sanidine feldspar FS08-VS grain mounts^396^
												Pakistan perthitic alkali feldspar FS06 thin section^396,397^
												Austrian sanidine K-feldspar FS07 (adularia) thin section^397^
Kiselev *et al*. 2021;^396^ PipeJet droplet generator	Printed droplet array (based on Peckhaus 2016)^384^	Capillary tube	Piezoelectric actuation	N/A	21.6 nl	70	None	None	N/A	3	Linkam MDBCS196 cryostage	Volkesfeld sanidine K-feldspar FS08-VS^396^
												Pakistan perthitic alkali feldspar FS06^397^
												Austrian sanidine K-feldspar FS07 (adularia)^397^
**Microcavity-based arrays**												
Häusler *et al*. 2018;^321^ “Freezing on a Chip”	Microcavity droplet array	Silicon or gold wafer	Microcavities	40 ± 4 nominal (20–80 range)	34 ± 11 nominal (4–300 range)	25	Paraffin oil	None	±0.4	2	TEC (water-ice cooled)	Purified water^321^
												K-feldspar microcline^321^
												Snomax®^321^
												*B. pendula* (silver birch) pollen washing water^321^
												*J. communis* (common juniper) pollen washing water^321^
Lee *et al*. 2023;^379^ “Nanoliter osmometer”	Microinjected droplet array (based on a nanoliter osmometer)^398^	Droplets injected into silver sample grid	Microinjector	∼280 to 350	∼10 to ∼20 nl	12	Unknown oil	None	N/A	2 nominal (0.5 and 1 also used)	TEC (water cooled)	Bacterial ice-nucleating proteins (from *P. syrinage* and *P. borealis*)^376,379^
												Snomax®^379^
												*E. coli* (ArcticExpress strain)^379^
												Phosphate buffered saline (PBS)^379^
												Miller's LB broth medium^379^
**Tubing-based arrays**												
Atig 2018^385^	Millifluidic spiral tubing-based array	PFA capillary (1 mm Ø)	Capillary-based T-junction or capillary-based flow focusing	∼800 to ∼1200	∼300 to ∼900 nl	∼70	3M^™^ Fluorinert^™^ FC-770 fluorocarbon or *n*-hexane, or cyclopentane as a hydrate-former	None	N/A	0.5	TEC (cooled via a cold bath)	Purified water^385^
												Dissolved CO_2_ in water^385^
												Montmorillonite clay^385^
												Titanium dioxide^385^
												Silver iodide^385^
Isenrich *et al*. 2022 ;^386^ “MINCZ”	Microfluidic serpentine tubing-based array	PDMS on glass with PFA capillary (75 *μ*m Ø)	Flow focusing	75 ± 5 or 100 ± 5	221 ± 44 or 524 ± 79	750	3M^™^ Novec^™^ 7500 (HFE-7500) fluorocarbon	1% v/v 008-FluoroSurfactant (RAN Biotechnologies)	±0.2	1	Ethanol bath cooled by a TEC (aq. ethylene glycol cooled TEC)	Purified water^386^
												Homogeneous freezing of water^399^
												Italian K-feldspar microcline^386^
												Sucrose^400^
**Continuous flow**												
Sgro *et al.* 2007^349^	Continuous flow	PDMS on glass	T-junction	30	14	N/A	Silicone oil (AS 4) or mineral oil (M5310)	0.01 or 0.05% w/v Span 80	N/A	∼20 000	TEC (air cooled)	*Biological cell freezing (mouse B lymphocytes)* ^349^
Stan *et al.* 2009^207^	Continuous flow	Glass on PDMS on glass	Flow focusing	80 ± 1	268 ± 10	>10 000	PFMD fluorocarbon	2% v/v THPFO	±0.4	120–6000	Series of TECs (ethanol cooled)	Purified water^207^
												Homogeneous freezing of water^207^
												Silver iodide^207^
												External electric fields^406^
												*Temperature-controlled droplet size/velocity* ^407^
												*Hydrodynamic droplet positioning* ^408^
												*Droplet lift forces* ^409^
Tarn *et al*. 2020;^203^ “LOC-NIPI”	Continuous flow	PDMS on glass	Flow focusing	86 ± 8	331 ± 89	1000s	3M^™^ Novec^™^ 7500 (HFE-7500) fluorocarbon	0.2 or 2% w/w Pico-Surf^™^ 1 (Sphere Fluidics)	±0.4 to ±0.7 (temperature dependent)	200–2400	TEC (aq. PPG cooled)	Purified water^203^
												Homogeneous freezing of water^322^
												Snomax®^203^
												*B. pendula* (silver birch) pollen washing water^203^
												Eastern Mediterranean aerosol^203^
												Canadian river water^410^
												*Continuous water/ice sorting* ^411^
Roy *et al.* 2021^206^	Continuous flow	PDMS on silicon	Flow focusing	70–85	221–322	1000s	Light mineral oil (CAS: 8042-47-5)	None	±0.03	140–720	Series of TECs (LN_2_ cooled)	Snomax®^206^
												Aged Snomax®^206^
												Heat treated Snomax®^206^

^a^ K-feldspar microcline BCS-376 is also known as “FS02” in some publications.

**Abbreviations:** aq. = aqueous; LN_2_ = liquid nitrogen; PDMS = poly(dimethyl siloxane); PFA = perfluoroalkoxy-alkane; PFMD = perfluoromethyldecalin; PFPE = perfluoropolyether; PPG = polypropylene glycol; Span 80 = sorbitan monooleate; TEC = Peltier element-based thermoelectric cooler (TECs must be actively cooled to achieve low temperatures and the cooling methods are described in brackets here); THPFO = 1*H*,1*H*,2*H*,2*H* -perfluorooctanol; Tris = tris(hydroxymethyl)aminomethane.

### A. Microfluidic droplet emulsions

The easiest and most accessible method of employing microfluidics for INP analysis is to pump an aqueous suspension of INPs or aerosol sample through a basic flow focusing or T-junction microchannel design to generate water-in-oil droplets and collect them off-chip in a vial as an emulsion (Fig. 5). Droplets can then be pipetted from the vial onto a standard microscope cold stage where they can undergo an otherwise traditional DFA [Fig. 5(c)]. In a stable oil-surfactant system, the aqueous droplets will self-assemble into a hexagonal close packed array that, despite the proximity of the droplets, allows the droplets to freeze independently.

Riechers *et al.*^367^ demonstrated the use of this method for the study of the homogeneous freezing of water using differential scanning calorimetry (DSC), in addition to cryomicroscope-based DFAs, to obtain high temperature accuracy, finding that temperature measurement is the most important parameter in the uncertainty of ice nucleation rates. At around the same time, Lignel *et al.*^368^ also performed DSC studies of microfluidically generated water droplets as part of a test on emulsion stability toward studies in microgravity conditions.

Weng *et al.*^369^ were the first to apply the technique to heterogeneous nucleation via INPs, focusing on cryopreservation studies. The authors tested Snomax®, a commercial form of the ice-nucleating bacteria, *P. syringae*, that has been sterilized and lyophilized, in water and heavy water (D_2_O), as well as testing the effect of several cryoprotectants on freezing.

Tarn *et al.*^201^ developed the “Microfluidic pL-NIPI” droplet emulsion technique to replace the previous pL-NIPI method that employed a nebulizer to generate droplets on a glass substrate. The original nebulizer technique yielded highly polydisperse populations with only minimal control over the droplet size and employed a liquid nitrogen cooled cryomicroscope stage,^370,371^ while the microfluidic version produced a highly monodisperse droplet population and employed a more user-friendly Peltier element-based thermoelectric cooler (TEC). The microfluidic pL-NIPI was used to assess a range of atmospheric INPs,^201^ including filter samples collected from a rural site and during bonfire events,^202^ and was also applied to Arctic sea surface microlayer (SML) and phytoplankton samples.^372^

These simple microfluidic devices require the use of otherwise standard cold stage equipment, which is an advantage of this technique, although it does require more steps than other microfluidic DFA methods. The number of droplets typically analyzed using this technique is on the order of hundreds to about one thousand, far higher than most standard DFAs. However, the stability of the droplet system tends to be lost once the droplets have been freeze-thawed, resulting in coalescence of the droplet population that prevents repeat freezing cycles from being performed. This method is also not so amenable to automation as other microfluidic methods, which would be an essential part of a sample-to-answer INP analysis platform.

### B. Microfluidic droplet arrays

**FIG. 6.**
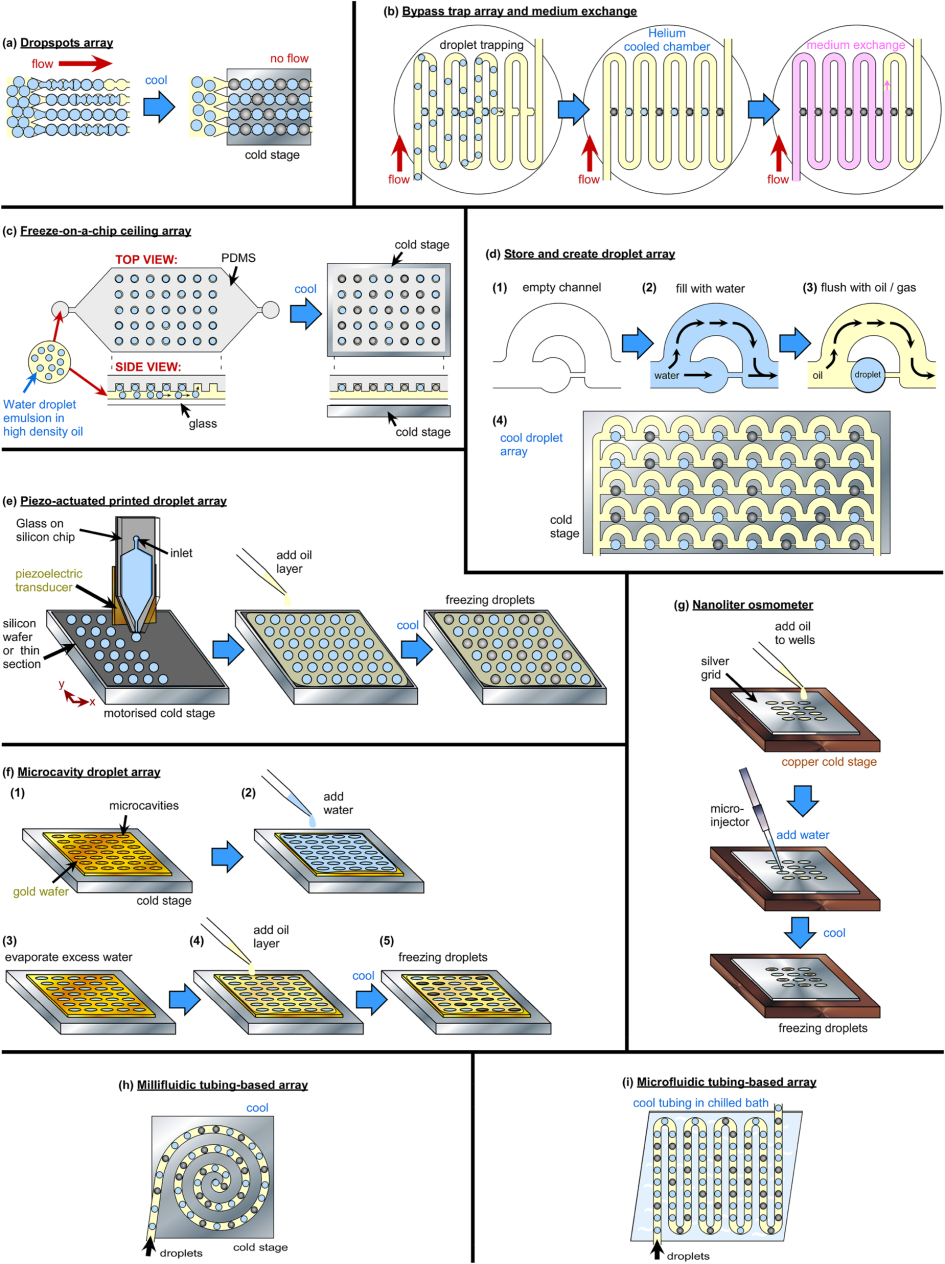
Microfluidic droplet array techniques for on-chip microfluidic DFAs, in which an array generated on a substrate is cooled to freezing temperatures. (a) Dropspots array technique,^373,374^ utilized also in the WISDOM^220^ and nanoBINARY^380^ DFA methods. Adapted and used with permission from Schmitz *et al.*, Lab Chip **9**, 44–49 (2009). Copyright 2009 Royal Society of Chemistry, permission conveyed through Copyright Clearance Center, Inc. (b) A bypass trap array for the exchange of the medium around frozen droplets, located in a helium cooled chamber.^381^ Adapted and used with permission from Sgro and Chiu, Lab Chip **10**, 1873–1877 (2010). Copyright 2010 Royal Society of Chemistry, permission conveyed through Copyright Clearance Center, Inc. (c) “Freeze-on-a-chip” ceiling array that relies on a high density oil to trap aqueous droplets in ceiling wells.^382^ Adapted and used with permission from Weng *et al.*, Cryobiology **84**, 91–94 (2018). Copyright 2018 Elsevier. (d) “Store and create” droplet array, in which water is flushed through a channel then flushed with oil^204^ (or backflushed with oil or gas)^383^ to leave droplets in traps, eliminating the need for surfactants as in many other techniques.^204^ Adapted and used with permission from Brubaker *et al.*, Aerosol Sci. Technol. **54**, 79–93 (2020), reprinted by permission of the publisher (Taylor & Francis Ltd, http://www.tandfonline.com). (e) Piezoeletric transducer actuated droplet printer used to automatically print a droplet array on a substrate on a motorised stage.^384^ (f) Microcavity-based “Freezing on a Chip” platform in which microcavities in a gold or gold-coated substrate are used to generate droplets in an array for freezing.^321^ (g) Nanoliter osmometer adapted for DFAs via the microinjection of droplets into oil-filled wells in a silver grid.^379^ (h) Millifluidic spiral tubing-based array on a cold plate.^385^ (i) Microfluidic serpentine tubing-based droplet array in a chilled bath.^386^

A more advanced form of the droplet emulsion technique involves the introduction of a microfluidic chamber or channel structure following the droplet generation region that enables the trapping of droplets in an array (Fig. 6). By situating the microfluidic chip directly onto a microscope cold stage, typically comprising a Peltier element-based TEC, the trapped droplet array can thus be cooled directly to perform a DFA. This allows for a much greater degree of automation than the droplet emulsion DFA technique by eliminating several manual steps, though it does require somewhat more complex chip design and fabrication.

Edd *et al.*^373^ developed the first instance of an on-chip array-based DFA using a “Dropspots” platform [Fig. 6(a)],^374^ which employs a series of parallel channels containing droplet-shaped wells. Droplets are generated and flow through the channels, then when the flow stops the droplets settle into the wells, allowing for a rapid and simple arraying process. The authors performed nucleation and crystallization experiments on water and aqueous solutions of glycerol.

The Dropspots technique was later developed into the INP analysis platform, WISDOM, by Reicher *et al.*,^220^ initially demonstrating its capabilities on nucleation of minerals and the analysis of atmospheric samples collected using a MOUDI cascade impactor during dust storms in the Eastern Mediterranean.^203,220,221^ WISDOM has since been applied to the study of various ice-nucleating materials such as sea ice diatoms,^32^ soil and mineral dusts,^317^ bacteria and proteins,^317,375–379^ and a variety of other samples and studies (see Table II for a complete list). A modified version of WISDOM, termed the “nanoliter Bielefeld Ice Nucleation ARraY (nanoBINARY),” was also recently applied to the study of ice nucleation by short- and long-chain poly(vinyl alcohol) (PVA).^380^

Sgro and Chiu^381^ developed a droplet docking device in which droplets would be pulled into bypass traps or “docks” and then frozen upon cooling, after which the immiscible environment around them was exchanged [Fig. 6(b)], though a DFA was not performed on any samples.

Weng *et al.*^382^ fabricated a “freeze-on-a-chip” DFA device comprising a series of wells in the ceiling of a microfluidic chamber for cryobiology studies [Fig. 6(c)]. Water-in-oil emulsions were generated microfluidically from their previously described device^369^ and then injected into the Freeze-on-a-chip, with the high density of the fluorinated oil causing the droplets to rise to the top of the device and become trapped in the wells. Around 1500 droplets could be trapped for DFAs of PVA as an antifreeze (glyco)protein mimic.

Brubaker *et al.*^204^ developed a DFA based on the “store and create” droplet microfluidic technique of Boukellal *et al.*^387^ that allows for the formation of droplets within wells in a parallelized microchannel structure [Fig. 6(d)]. Aqueous suspension is first pumped through the channels to fill the wells, and then the channel is flushed with oil that removes the aqueous suspension but avoids the wells, resulting in the *in situ* surfactant-free generation of 6 nl water-in-oil droplets within the wells. The technique was applied to DFAs of NX illite, Snomax®, and filter-collected biomass-burning aerosol (BBA),^204^ including the finding that atmospheric aging enhances the ice nucleation of BBA,^205^ and as part of a study on interferences in purified water freezing in DFAs.^328^ Since the droplets were relatively large, the authors refrained from labeling their purified water data as homogeneous freezing, but decreasing the droplet well size could easily achieve this in future.

“Store and create” devices have also now been adopted by other research groups. Roy *et al.*^388^ employed such a platform for the analysis of INPs and efflorescence in SSAs from bulk seawater and sea surface microlayer (SML) samples, finding that the droplets that effloresced into aggregate and amorphous particles correlated with warmer droplet freezing temperatures during DFAs, while those that effloresced into single and fractal crystals correlated with colder freezing temperatures. House and Dutcher^389^ studied the effects of cationic salts on the ice-nucleating ability of Snomax® as a seawater proxy and the final morphology of the particles, finding a decoupling of ice-nucleating activity and particle morphology. House and Dutcher^390^ later studied the effects of salinity and pH on montmorillonite bentonite clay, in addition to repeat freezing and efflorescence–deliquescence (E–D) cycling. The results showed that the ice-nucleating ability of montmorillonite decreased at low pH, possibly due to changes in particle aggregate sizes, while E–D cycling affected the freezing characteristics of the suspensions, which may be due to delamination of the clay particles. The group has also used “store-and-create” platforms for investigations into phase transitions such as crystallization and liquid–liquid phase separations,^352^ including in aerosols and SMLs.^391–393^

Tarn *et al.*^383^ demonstrated a store and create array device that allowed for the generation of droplets onto polished mineral thin sections to map the ice-nucleating activity across the mineral surface, in an update to the single droplet version of the technique used by Holden *et al.*^394^ to study ice-active sites. The device used dried air or from the channels nitrogen gas to backflush the device (rather than flushing in the same direction as the original water fill, as per Brubaker *et al.*^204^) in a similar method to Kim *et al.*^366^ who used store and create for inorganic crystallization. The use of dry air or nitrogen eliminated the need for oil, while the technique could be operated by hand without the need for syringe pumps.

### C. Printed droplet arrays

A different method of generating an array of picoliter droplets is to print them directly onto a substrate [Fig. 6(e)]. Peckhaus *et al.*^384^ developed a system based on a commercially available piezoelectric microfluidic droplet generator (GeSiM, Germany) and a motorized cold stage, allowing up to 1500 picoliter droplets to be printed automatically onto a silicon wafer that was then covered with a layer of oil. The printed array was used to analyze suspensions of ice-nucleating feldspars^384^ and alumina,^395^ as well as being used to array directly onto polished grain mounts and thin sections of minerals.^396,397^ The group used a similar approach to print larger droplets (21.6 nl) via a piezo-driven PipeJet Nano dispenser (BioFluidix GmbH) onto silicon wafers for DFAs of feldspar suspensions.^396,397^

### D. Microcavity-based arrays

Droplet arrays have been demonstrated using microcavities or wells fabricated in a substrate into which droplets can be placed or generated. Häusler *et al.*^321^ developed a “Freezing on a Chip” technique comprising a gold-plated silicon or gold substrate into which an array of wells was etched [Fig. 6(f)]. 2 *μ*l of an aqueous INP suspension was pipetted onto the chip to fill the wells, with the excess liquid between the wells evaporating, then the wells were covered in a layer of oil. The droplet size was determined by the size of the wells, and multiple chips of different well sizes were fabricated to accommodate different droplet volumes. The device was applied to DFAs of Snomax®, pollen, and feldspar mineral.

Lee *et al.*^379^ developed a DFA based on a commercially available nanoliter osmometer (*μ*Ice, Israel), originally developed for single droplet freezing studies by Braslavsky and Drori,^398^ that comprises 12 oil-filled wells of 0.5 mm diameter into which ∼10 nl aqueous droplets were added using a FemtoJet microinjector (Eppendorf, Germany) [Fig. 6(g)]. The device was applied to DFAs of ice-nucleating proteins from *P. borealis* bacteria to study their self-assembly, alongside DFAs performed using WISDOM for comparison.^376,379^

### E. Tubing-based arrays

Recently, microfluidic platforms have been developed that bridge the features of the droplet emulsion systems and the droplet array devices described above. Here, water-in-oil droplets are generated in a microfluidic channel and enter a very long section of spiraled or serpentine transparent tubing. However, rather than being collected in a vial for off-chip analysis, the flow is instead stopped such that the droplets in the tubing become stationary, forming a tubing-based droplet array that can be cooled to perform a DFA. This methodology combines simple fabrication and setup with excellent temperature control over the droplet array and could be automated relatively easily.

Atig *et al.*^385^ fabricated a millifluidic capillary tubing-based T-junction droplet generator that fed into a spiral capillary immersed in an ethanol cold bath [Fig. 6(h)]. DFAs of montmorillonite clay, titanium dioxide, and highly ice nucleation active silver iodide were performed, though the droplets were on the scale of millimeters in diameter that yielded high background results for purified water DFAs.

Isenrich *et al.*^386^ developed a microfluidic tubing array-based device, the Microfluidic Ice Nuclei Counter Zürich (MINCZ), in which microfluidically generated droplets were stored in capillary tubing held within a plastic holder with temperature probes and immersed in an ethanol cooling bath [Fig. 6(i)]. The platform was used to perform DFAs on purified water,^399^ K-feldspar,^386^ and aqueous sucrose solutions^400^ with temperature accuracies of ±0.2 °C.

### F. Continuous flow analysis

Continuous flow DFAs comprise a cold plate directly beneath a long microchannel such that droplets freeze as they flow over the plate. This has the advantage that, unlike most other DFAs that are limited to tens to hundreds of droplets per experiment, thousands or tens of thousands of droplets can be assayed by allowing the platform to run for as long as desired. Continuous flow microfluidic systems are also very amenable to automation, and a raft of options are available for upstream or downstream processing including continuous flow separations,^291^ reactions and sample treatment,^401,402^ and analysis.^403,404^

However, maximizing the potential for this technology can also lead to complexity and a potential for issues to arise. These include the need for accurate temperature measurements of the flowing droplets within the microchannel^355^ without disturbing the droplets, which could potentially trigger freezing. Microchannel dimensions become very important: a small microchannel cross section relative to the droplet size is desirable to limit the temperature differences through the cross section and so improve heat transfer, but must be larger enough that the droplets do not become stuck when they freeze due to the ∼9% increase in volume (depending on temperature), or when spicules of ice extrude from a frozen droplet.^405^

While droplets can easily be generated at rates of hundreds to thousands per second, the flow rates required for this while still being able to freeze droplets would require very low cold stage temperatures and extremely high temperature gradients that would be far greater than the gradients the droplets would experience in updrafts in the atmosphere. Therefore, continuous flow DFAs would be limited to single digits to tens of droplets per second without incorporating measures to lower the temperature gradient, e.g., the use of serpentine channels. Despite these issues, continuous flow DFAs arguably offer the greatest potential for integrated lab-on-a-chip platforms with upstream and downstream processing.

Sgro *et al.*^349^ demonstrated the first example of continuous flow droplet freezing, albeit for cryopreservation studies of cells rather than as a DFA, in 2007. Using a Peltier element-based TEC either above or below the microchannel, droplets containing single cells were frozen in flow and found to be viable provided cryoprotectants (in this case, dimethyl sulfoxide, DMSO) were present.

**FIG. 7.**
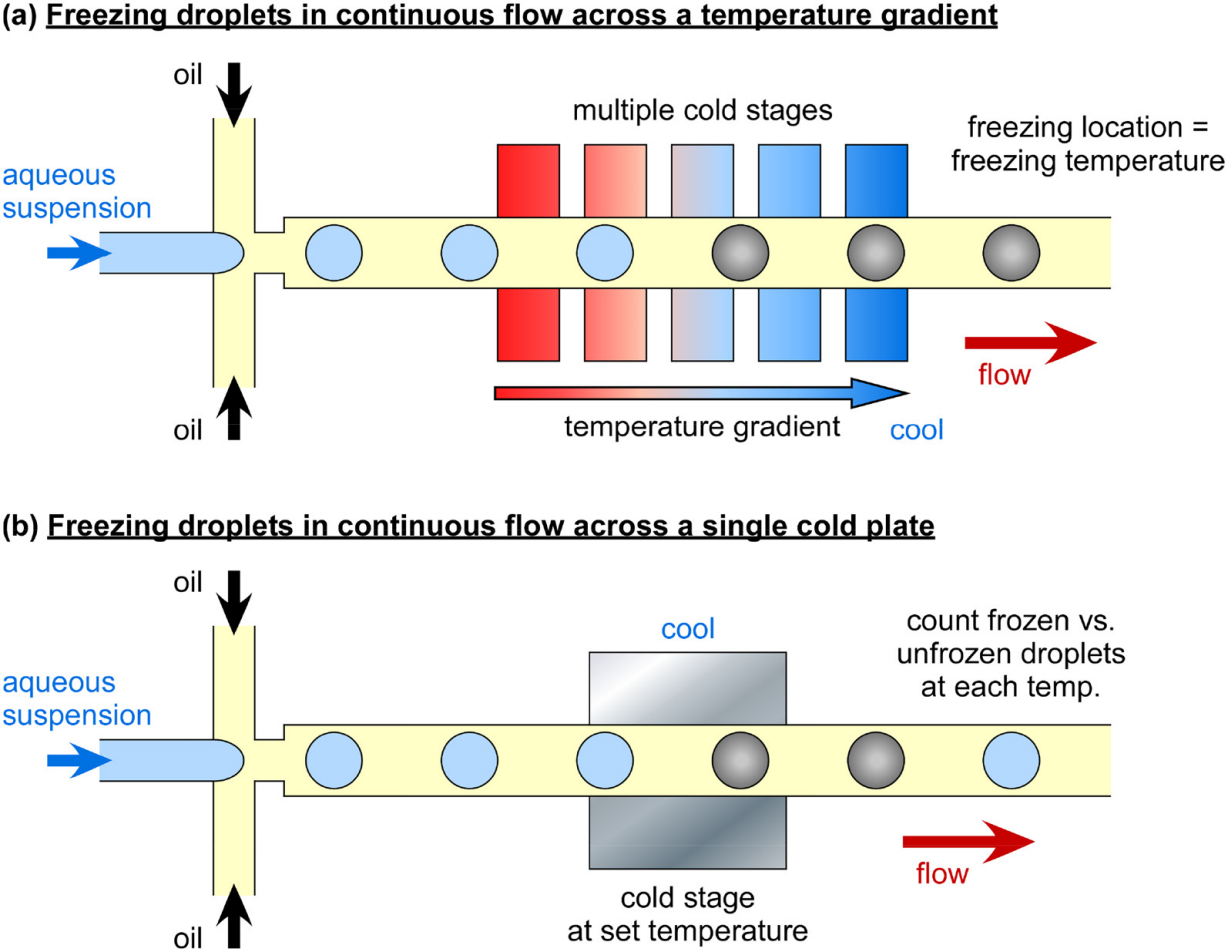
Continuous flow microfluidic DFAs, in which droplets are generated and then freeze as they pass over a cold stage, allowing the analysis of thousands of droplets. (a) Use of a multi-cold stage instrument to generate a defined temperature gradient.^207^ The position at which a droplet freezes thus indicates the temperature at which it froze. (b) Use of a single cold plate, in which the relative number of frozen and unfrozen droplets are counted over a series of set temperatures of the stage, as used in the LOC-NIPI platform.^203^ Adapted from Tarn *et al.*, Lab Chip **20**, 2889–2910 (2020). Copyright 2020, Author(s) licensed under a Creative Commons Attribution 3.0 Unported License.

Stan *et al.*^207^ fabricated an elegant continuous DFA platform featuring a series of thermoelectric coolers that generated a temperature gradient along the length of the microchannel [Fig. 7(a)]. A series of microfabricated platinum resistance temperature (PRT) detectors were integrated into the bottom of the microchannel, such that the temperature at which the droplets froze could be determined based on their position in the temperature gradient system. A platinum coating on the underside of the device served as a mirror to aid visualization using reflected light microscopy. The apparatus allowed tens of thousands of droplets to be analyzed at a rate of 75 droplets per second, with high temperature accuracy (±0.4 °C). DFAs of purified water and silver iodide were demonstrated, while the dendritic growth of ice in flowing droplets was studied with high-speed microscopy.

The instrument of Stan *et al.* was modified to determine whether electric fields influence the homogeneous nucleation of supercooled water by incorporating electrodes above and below the microchannel.^406^ The authors found that applying electric fields up to 1.6 × 10^5^ V m^−1^ had no effect on nucleation rates, though thermodynamic models suggested that fields >10^7^ V m^−1^ could increase the rate of nucleation. Variations of the platform were also employed to demonstrate the effect of temperature on controlling droplet size and velocity,^407^ and investigations of sheathless hydrodynamic positioning^408^ and lift forces^409^ on droplets and bubbles flowing through a microchannel.

Tarn *et al.*^203^ developed the continuous flow LOC-NIPI platform that comprised a single cooling plate, similar to Sgro *et al.*,^349^ and performed DFAs by flowing droplets over the plate at a series of decreasing temperatures [Fig. 7(b)], with hundreds or thousands of droplets analyzed per temperature setpoint. Modeling of the flow and temperature was not required, and a temperature probe was located in a parallel reference channel to measure the temperature of the flowing oil as a proxy for droplet temperature measurements. The temperature gradient across the single cold plate varied with temperature, which, combined with the use of the temperature reference channel rather than direct measurements as in Stan *et al.*,^207^ yielded conservative temperature uncertainties that ranged from ±0.4 °C at warmer temperatures to ±0.7 °C at the coldest temperatures.

The LOC-NIPI setup allowed for non-specialists in microfluidics to use the setup in the lab and in the field, being applied to DFAs of Snomax®, birch pollen, aerosol filter samples collected and analyzed in the Eastern Mediterranean,^203^ INP activity in river outflows,^410^ and a study on homogeneous nucleation.^322^ LOC-NIPI was designed to be built into an integrated analysis platform with upstream and downstream processing, the first step being an adaptation to sort ice crystals and water droplets in continuous flow,^411^ described in Sec. V.

An issue with continuous flow DFAs is the sheer number of droplets that pass through the device and must be counted, an extremely laborious job if performed manually. Roy *et al.*^206^ fabricated a continuous flow DFA platform based on that of Stan *et al.*^207^ and developed a deep neural network (DNN) algorithm using AlexNet^412^ to count droplet freezing events with 99% accuracy that was applied it to the study of the effect of heat treatment on Snomax®. Using Fourier-transform infrared (FTIR) spectroscopy, the authors showed that heat treatment causes the β-helix secondary structure of Snomax's *inaZ* protein to convert to a β-sheet or strand-like structure and that the extent of β-helix conversion correlated with a reduction in droplet freezing temperatures in the microfluidic device. The platform was limited to temperatures down to −20 °C due to the use of mineral oil, which becomes too viscous to pump through the microchannel, but this could easily be overcome by using similar oils to other continuous flow DFA systems.

A further potential issue with continuous flow DFAs is the typically very high cooling rate (hundreds to thousands of °C min^−1^), much faster than typical cooling rates experienced in cloud updrafts (e.g., 1–10 °C min^−1^) that are more accurately represented in droplet emulsion or array-based DFAs, i.e., static rather than continuous systems. However, a comparison of LOC-NIPI data obtained for silver birch pollen (*B. pendula*) and Snomax® demonstrated that even at very high cooling rates of 2400 °C min^−1^, the data were comparable to other standard DFA techniques performed at 1 °C min^−1^.^203^

While it has not been demonstrated yet, the microfluidic tubing array DFA platforms described in Sec. IV E, such as MINCZ,^386^ could easily be adapted to continuous flow analysis by having droplets flow through tubing immersed in a bath that is being cooled down while the droplets are observed.

## V. MICROFLUIDIC DROPLET SORTING

One of the most challenging aspects of atmospheric ice nucleation, as discussed earlier, is the identification of the dominant INP species in an atmospheric population. While there are bioanalytical techniques that can be used to determine the presence of biological INPs, it may be necessary or beneficial to separate the dominant INPs, which trigger freezing in DFAs at warmer temperatures, from the “background” INP community that triggers freezing at colder temperatures, to determine what species are in the former (and in what concentrations) that are not in the latter. This can be achieved somewhat, for example, by using laborious manual processes in which droplets that freeze at warmer temperatures in a DFA are repeatedly selected, divided, and refrozen multiple times, then the final droplet evaporated, and the residual analyzed or photographed.^109^

**FIG. 8.**
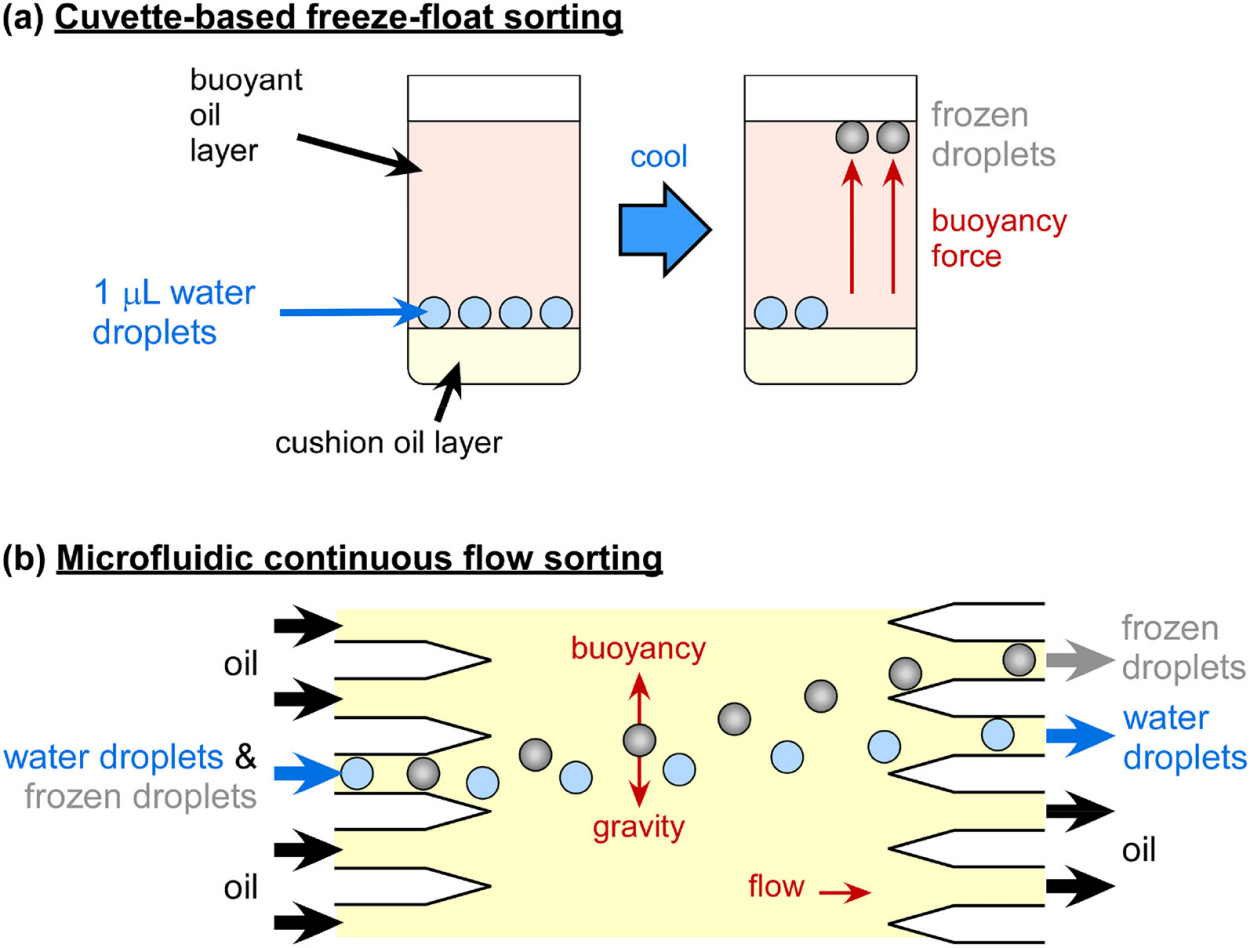
The sorting of frozen and unfrozen droplets for the later analysis and comparison of the two populations, based on the greater density of water to ice. (a) Freeze-float sorting in a cuvette in a non-microfluidic method, utilizing oils of differing densities to generate cushion and buoyancy layers.^415^ Reprinted (adapted) with permission from Kamijo and Derda, Langmuir **35**, 359–364 (2019). Copyright 2019 American Chemical Society. (b) Microfluidic continuous flow sorting of frozen and unfrozen droplets based on their relative buoyancies under gravity, allowing the collection of the two populations via different outlets, as used in the LOC-NIPI platform.^411^ Adapted from Porter *et al*., Lab Chip **20**, 3876–3887 (2020) Copyright 2020, Author(s) licensed under a Creative Commons Attribution 3.0 Unported License.

Fahy *et al.*^413^ recognized the potential for the density-based separation of frozen and unfrozen droplets (*ρ*_water_ > *ρ*_ice_) after finding that ice crystals formed in an aqueous solution of 50% w/w propylene glycol floated to the top of the solution.^414^ Kamijo and Derda^415^ developed a cuvette-based “freeze-float” droplet selection system for 1 *μ*l droplets suspended in layers of oils of differing densities, with single droplets finding equilibrium positions in the different layers depending on whether they were frozen or not, allowing for their collection [Fig. 8(a)]. The authors have since demonstrated a high-throughput version of the platform utilizing multiwell plates and a robotic liquid handling system for automated pipetting of droplets.^416^

Porter *et al.*^411^ demonstrated a density-based continuous flow microfluidic sorting system for frozen and unfrozen droplets by adding a separation chamber to the LOC-NIPI platform [Fig. 8(a)]. Droplets and ice crystals entered the separation chamber in a high density oil such that both populations rose upwards (in the z-direction) in the oil against the force of gravity, with the less dense and therefore more buoyant ice crystals migrating further in the z-direction and thus being collected via a different outlet channel to the water droplets. A separation efficiency of 94% was achieved, with scope for improvement by modifying the design.

The device of Porter *et al.*^411^ is currently the only microfluidic platform to achieve an ice crystal–water droplet separation, but various continuous flow separation systems discussed in Sec. II could also be applied here. Size-based separations could be feasible in principle, though the increase in volume by ∼9% of a droplet upon freezing may be too small to make this easily achievable.

However, deformability-assisted sorting methods could be employed to separate the deformable water droplets from the solid ice crystals, and these methods have seen success in the separation of biological cells.^417–419^ Such techniques can utilize microstructures, e.g., pillars or weirs, that the deformable species can flow under/over/around in a manner that solid particles cannot, or hydrodynamic forces (including inertial microfluidics) that leverage the hydrodynamic resistances of different species in fluids to force them into different laminar flow streams. Acoustophoretic forces,^420^ applied via ultrasonic transducers, enable microfluidic separations based on size, density, and compressibility to manipulate particles into equilibrium positions in a microchannel and could have potential applications here. Deformation cytometry techniques, often applied to single cell analysis of cell biomechanical properties, could also be applied here by exploiting the differences in mechanical stiffness to manipulate and separate droplets and crystals.^419^

A key point to consider here, however, is that the mechanism of separation does not induce the freezing of water droplets prior to the sorting outlets.

## VI. DROPLET PICOINJECTION

Following the DFA and potentially the droplet sorting steps, an ideal on-chip automated platform would incorporate various types of chemical and biological analysis, such as immunoassays, colourimetric or fluorimetric reactions, or DNA analysis. Many such measurements require the mixing of reagents with the sample in order to react with the analytes of interest, but since samples are compartmentalized in droplet microfluidics, the interfacial tension between the droplets and the immiscible oil can make this difficult to achieve.

**FIG. 9.**
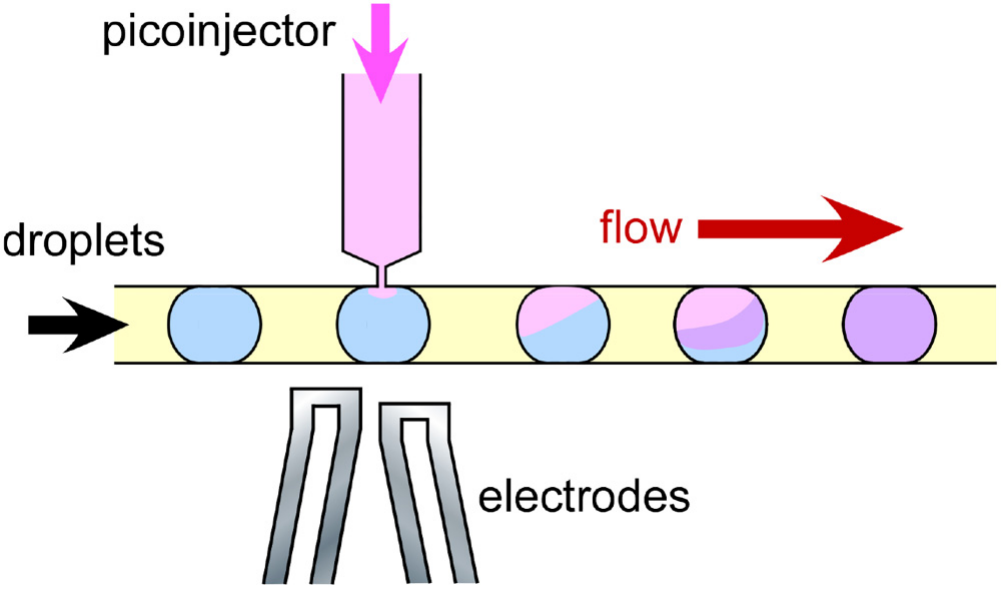
Picoinjection of biochemical reagents into droplets as they flow past a narrow channel picoinjector.^421^ Reprinted (adapted) with permission from Abate *et al.*, Proc. Natl. Acad. Sci. U. S. A. **107**, 19163–19166 (2010). Copyright 2010 National Academy of Sciences. The interface between the aqueous droplet and the surrounding immiscible oil is momentarily perturbed, allowing injection. The perturbation is often achieved by applying an electric field via microelectrodes, though the use of controlled pressure or the Venturi effect can also be applied.^435^ Picoinjection of reagents can allow downstream biochemical analysis to be performed, such as single cell analyses, immunoassays, or DNA analysis.

One of the most common methods of injecting picoliter reagents into flowing microfluidic droplets utilizes integrated electrodes to electrically induce a thin-film instability that momentarily ruptures the water–oil interface, allowing reagent from a narrow side-channel under high pressure to be injected into the droplets (Fig. 9). This technique was first demonstrated by Abate *et al*.^421^ and has since been applied to the injection of reagents for DNA amplification,^422,423^ single cell lysis,^424^ microgel bead fabrication,^425,426^ and variations on the electro-injection method.^423,427–429^ The technique has also been applied to droplet merging to enable the merging and reaction of biochemical species in two different droplet populations.^430–432^

In many situations, it may be desirable to not use electrodes within a device, hence electrode-free picoinjection methods have also been developed. O’Donovan *et al*.^433^ developed a method in which dissolved electrolytes in the solution acted as the electrode, allowing picoinjection when an electric field was applied. Yuan *et al*.^434^ demonstrated a truly electrode-free system in which picoinjection was achieved by finely controlling the pressures in the microfluidic device, with the picoinjection microchannel actuated by air pressure controlled via a regulator. Li *et al.*^435^ exploited the Venturi effect via a narrow hydrophilic microcapillary junction that injected reagents as droplets interacted with the capillary as they flowed past. Niu *et al.*^436^ fabricated a pillar-based platform that slowed droplets and forced the succeeding droplet to merge with the slowed droplet under pressure.

Picoinjection is a powerful droplet manipulation method that is crucial for many subsequent downstream biochemical analyses, with several methods available depending on requirements.

## VII. MICROFLUIDIC BIOAEROSOL ANALYSIS

Microfluidic technology has a number of features that have made it particularly applicable to bioanalysis.^437,438^ The reduction in device size brings with it the ability to handle small amounts of potentially precious sample volumes and reduces the amounts of expensive reagents consumed. The small volumes of microchannels reduce the diffusion distances between sample and reagent molecules, which, combined with a myriad of potential mixing techniques, enable rapid reactions and assays. Large surface-to-volume ratios provided by microchannels allow for faster heating and cooling, and microfabrication technologies allow for the integration of miniaturized temperature control^355,439^ and detection^440^ systems.

Droplet microfluidic (or digital microfluidic) systems employ monodisperse droplets that can be used as thousands of identical, highly efficient picoliter reaction vessels that can be manipulated and analyzed on a droplet-by-droplet basis, allowing for powerful, high throughput bioanalytical processing (e.g., single cell analysis, immunoassays, and DNA analysis).^334^ These features, combined with the potential for small footprint, portable devices, have made microfluidics an attractive technology for point-of-care medical devices,^441^ for example. Likewise, microfluidic techniques have been applied to a variety of bioaerosol separation and analysis procedures, and reviews dedicated to this subject are available courtesy of Zhang *et al.*,^177^ Ezzre *et al.*,^182^ Lee *et al.*,^179^ Wang *et al.*,^178^ and Huffman *et al.*^181^ Related to the topic of ice nucleation, Zhao and Fu^442^ reviewed the use of microfluidics for cryopreservation, including cell manipulation, cryoprotective agent exposure, programmed freezing/thawing, vitrification, and *in situ* assessment in cryopreservation, and some of those processes may be applicable to microfluidic INP analysis.^442^

Given the broad scope of microfluidic bioanalytical techniques, and with dedicated reviews available elsewhere for each topic, here we provide a brief overview of some of the major techniques that could be applied to an automated INP monitoring platform, many of which could be used in conjunction with the picoinjection technique described above.

### A. Heat test for proteinaceous INPs

As described in Sec. I, the heat test is one of the most common techniques applied to the indirect determination of potential proteinaceous INP content.^13,59,134^ This is based on the principle that heat (e.g., 95 °C for 30 min) will denature^443^ an ice-nucleating protein and so reduce its ice-nucleating activity when comparing DFAs before and after the treatment. Heating to 95 °C in a microfluidic platform is easily achievable via a number of methods,^355,439^ though 30 min is a long timeframe for an envisaged automated analysis system. However, much as the confined nature of microfluidic devices make them amenable for deep supercooling for DFAs, they can likewise be used to achieve superheating of water (i.e., temperatures >100 °C without boiling) in the absence of nucleation sites in microchannels^444^ and droplets.^445^ Indeed, microfluidic superheating has been applied to the rapid breaking of spores^444^ and decomposition of peptides^446^ and proteins^447^ in continuous flow within a matter of seconds for their subsequent analysis.

While rapid heat test treatment is possible in a continuous flow microfluidic device, the test itself has a number of caveats in its interpretation. In particular, some minerals (such as quartz) also exhibit a loss of ice-nucleating activity upon heating, in addition to proteinaceous materials, while the mineral K-feldspar does not appreciably lose activity.^134^ Hence, if the ice-nucleating activity of a mineral population is dominated by K-feldspar, then the heat test can be used to represent the presence of proteinaceous INPs; otherwise, a loss of ice-nucleating activity could be due to either mineral content or proteinaceous materials. The heat test is also not suitable for all types of biological and biogenic INPs, for example, those whose ice-nucleating activity is conferred by polysaccharides or other non-proteinaceous means. More direct analytical methods, such as many of those described below, are therefore more attractive for ensuring the identification and quantification of the presence of biological and biogenic INPs.

### B. Chemical tests for INPS

Similar to heat tests, several simple chemical tests exist that can be routinely applied to INP analysis that provide indirect means of possible classification.^125^ For example, treatment with hydrogen peroxide diminishes the activity of organic INPs, including biological INPs, via an oxidation reaction, while guanidinium chloride treatment denatures bacterial and fungal proteins. Lysozyme affects ice-nucleating bacteria via the hydrolysis of peptidoglycan in the cell walls but may underestimate Gram-negative bacteria and also affects feldspar. Sometimes, several of these tests are performed on the same samples, such as heat tests and peroxide treatments, to determine the fractions of different types of materials present in an INP population, e.g., the heat-labile proteinaceous fraction vs the heat-resistant bio-organic fraction vs the mineral fraction.

Each of these treatments could be readily applied to a microfluidic platform via, for example, picoinjection of the chemicals into INP-containing droplets, including in parallel, with DFAs performed before and after the treatment. However, like the heat test, there are more direct bioanalytical procedures that can be applied to the identification and quantification of biological INPs.

### C. DNA analysis

Typically, an air particulate sample comprises hundreds or thousands of different biological particles, containing relatively few species in high abundance, a few tens of species with moderate abundance, and dozens or even hundreds of other species in very low abundance.^448^ DNA analysis is a powerful tool that allows for identification and quantification of biological species in a sample, including INPs, with a number of strategies available depending on requirements. This generally follows several key steps: extraction of DNA, amplification of specific genes or sequences, and analysis of the amplified DNA.

DNA sequencing of a sample allows for species identification and the determination of community composition and relative abundance. 16S rRNA^449^ and ITS^450^ sequencing have been employed during INP analyses for bacteria^135^ and fungal^451^ populations, respectively. Commercially available instrumentation, such as nanopore sequencing,^452^ has been readily demonstrated for in-field applications such as environmental DNA analysis.^453^ One challenge is that a significant number of genetic sequences remain unknown, for example, only around 1% of the estimated 2.2–3.8 M species of fungi have actually been sequenced;^454^ hence, it can be difficult to assign a detected sequence to a species.

While sequencing is not specific for INPs and requires the assignment of detected sequences to known species, other forms of DNA analysis enable the direct identification of known INP species. This is one of the more commonly applied biological measurements for INPs by groups with the requisite expertise. There are various methods of nucleic acid analysis that can provide useful information depending on the particular application. A simple presence/absence analysis for specific biological species, for example, *P. syringae*, can be achieved using traditional PCR techniques, in which amplification of the DNA is achieved by thermocycling of the extracted nucleic acids followed by gel electrophoretic analysis or fluorescence detection. The presence/absence tests also lend themselves well to the use of LAMP techniques. LAMP assays are highly sensitive, do not require precision thermal cycling instrumentation, and can be monitored using a variety of detection techniques including easy to interpret color change reactions.

However, neither sequencing nor presence/absence assays would, by themselves, inform on the ice-nucleating ability of the species present. To achieve this, it is necessary to identify and potentially quantify the *ina* gene that encodes the ice-nucleating proteins. Identification of *ina* can be achieved via PCR, though the gene can be detected in culturable, viable but non-culturable (VBNC), moribund, and dead cells. Further, detection of the *ina* gene also does necessarily indicate an efficient INP since the high nucleation efficiency of bacteria is typically conferred by the aggregation of proteins, which PCR cannot distinguish.

A modified version of the PCR technique, known as quantitative PCR (qPCR),^455^ provides information not only on the presence of the *ina* gene but also to what extent it is present. While allelic variation exists in the *ina* gene, it is possible to design qPCR primers that are able to amplify all known variants by targeting conservated sequences.^63^ Variations of traditional qPCR include digital qPCR, which separates the sample into microscopic droplets that each contain PCR reagents, and microarrays that allow for the targeting of different sequences in each well of the array, enabling multiplexed identification and quantification of several species simultaneously.

Microfluidic devices for the analysis of nucleic acids have become popular in many fields, particularly for clinical diagnostics, due to short reaction times and rapid heating/cooling times afforded by miniaturized samples and reagent volumes.^456,457^ While not yet fully explored for the analysis of INPs, there is great potential for these techniques to be adapted in this field for the detection and characterization of biological components. Compared to conventional nucleic acid analysis, microfluidics is particularly advantageous in enabling the integration of the multiple steps required, commonly cell lysis, nucleic acid extraction, amplification, and detection, with high sensitivity.

The field of microfluidic nucleic acid analysis is vast and the reader is directed to comprehensive reviews of the common individual elements, DNA extraction, LAMP,^458^ PCR and detection,^458^ as well as integrated analysis^459^ for further details. The use of a microfluidic LAMP assay for bioaerosols has already been demonstrated for the rapid detection of *P. aeruginosa*, an ice-nucleating bacteria studied in this instance as a multidrug resistant pathogen, collected via on-chip SHM-based sampling in a sample-to-answer platform.^216,288,289^

Microfluidic digital DNA analysis^460,461^ is particularly amenable to integration into an on-chip INP measurement platform given that droplets are required for the DFA step and that reagents such as primers can be introduced into each droplet via picoinjection. This strategy brings with it the capability for single cell analysis.^462^ Likewise, microfluidic DNA microarray analysis can also be exploited.^463^ However, despite the huge potential offered by microfluidic approaches to examining nucleic acids for INP analysis, there remain some key challenges.

First, a key issue for long-term monitoring is that of reagent storage, with the enzymes used for DNA amplification being particularly temperature sensitive, though this could be addressed using lyophilization of reagents, as has been demonstrated for both PCR^464^ and LAMP^465^ reagents.

Second, while sequencing is a very powerful method, it generates a large amount of data that must be processed using bioinformatics techniques, which can require considerable scientific expertise. While sequencing itself could be performed relatively quickly and easily on-chip, the amount and complexity of the data produced may be the main drawback to its use in long term atmospheric monitoring. However, more focused DNA analytical techniques would be far more amenable to long term automated monitoring in the field, for example, the use of LAMP or qPCR for identification and/or quantification of the *ina* gene or specific species.

### D. Cell culture and colony counting

The culturing of cells and subsequent counting of colony forming units (CFUs) has been performed on fungal and bacterial colonies from samples collected during INP measurement campaigns, including the testing of the ice-nucleating activity of the cultures.^56,140^ Culturing typically takes days to weeks, hence culturing and colony counting is not particularly amenable to sample-to-answer platforms.

However, the culturing of mammalian^466^ and bacterial cells^467^ in microfluidic devices is now routine, with fields such as organ-on-chip^468^ and bacterial biofilm models^469^ becoming increasingly popular. Microfluidic environments enable spatial and temporal control over the cells alongside continuous replenishment of culture media and *in situ* monitoring and analysis of the cells. Microfluidic single cell analysis is likewise routine,^470,471^ with droplet-based methods being very powerful,^472^ and amenable to a range of detection techniques described here (e.g., fluorescence staining, immunoassays, and DNA analysis). Hence, there are a number of opportunities for cell culture or single cell analysis for the microfluidic study of biological INPs.

Traditional off-chip cell culturing and CFU assays have also been applied to the analysis of bioaerosols collected using microfluidic sampling techniques. The SHM-based microfluidic sampler of Jing *et al.*^242^ was applied to the capture and CFU analysis of *E. coli* and *Mycobacterium smegmatis*. The spiral SHM of Bian *et al.*^290^ was employed for *Vibrio parahaemolyticus*, *Listeria monocytogenes*, and *E. coli*, while the continuous Dean flow impinger of Choi *et al.*^235^ was applied to the analysis of *S. epidermidis*.

### E. Immunoassays

Immunoassays are a powerful bioanalytical tool that allows for high sensitivity and specificity in the detection of biological species via an antigen–antibody interaction.^473^ Typically, the target analyte (antigen), such as a bacterial cell or protein, is captured by an antibody specific for that analyte, allowing it to be processed in a number of ways including its isolation from a sample matrix and detection via techniques such as fluorescence, colorimetric assays, or electrochemical sensors.

Immunoassays are traditionally performed in multi-well plates and typically require multiple laborious and manual processing steps but are highly amenable to microfluidics thanks to the rapid reactions and high surface-to-volume ratios available. Immunoassays have become one of the most routinely employed bioanalytical techniques in microfluidic technology,^474–477^ including for automated systems and point-of-care diagnostics,^160^ most commonly via heterogeneous assays that occur on antibodies bound to surfaces such as channel walls or microparticles.^474^ Magnetic particles find extensive use in microfluidic immunoassays thanks to the ability to manipulate them using internal or external magnets, allowing easy extraction of target analytes from samples and for sequential reactions to be performed.^478^ The integration of electrochemical and optical sensors into microfluidic devices enables the detection of the extracted and labeled analytes.^440^

Microfluidic immunoassays have successfully been applied to bioaerosol analysis. Jing *et al.*^287^ employed SHM-based bioaerosol capture of *Mycobacterium tuberculosis* on-chip, followed by elution and introduction into a second microfluidic device for bacterial lysis and analysis via a microparticle-based immunoassay with fluorescence detection. Coudron *et al.*^479^ developed an EWOD-based immunoassay platform for the automated analysis of *E. coli*, *Bacillus atrophaeus*, and MS2 bacteriophage via magnetic particle-based extraction and chemiluminescence detection. While the platform was not applied to bioaerosol analysis, it was proposed to be used in conjunction with the ESP sampler and EWOD droplet system developed by Foat *et al.*^239^

While immunoassays have not been performed for INP analysis to our knowledge, the assay of known biological INPs is possible via commercially available antibodies for those targets, such as *P. syringae*.^480^ However, since different strains of the same species can have varying or no ice-nucleating ability, the presence of the target analyte would not necessarily identify it as an INP. In this case, targeting known ice-nucleating proteins (e.g., *inaZ* protein) would provide a direct means of identifying and quantifying biological ice-nucleating activity. However, this depends on the availability of suitable antibodies, which are currently limited, or the capability to produce them.

### F. Raman spectroscopy

Raman spectroscopy is a vibrational spectroscopy technique that allows for chemical identification and quantification via structural fingerprints. While by no means routinely applied to INP analysis, it has been used to characterize ice residuals following DFAs in terms of organic matter, nitrates, sulphates, carbonates, and clay minerals,^481–483^ and measuring changes in ice-nucleating materials such as Snomax® following their chemical treatment.^484^

On-chip Raman spectroscopy and surface-enhanced Raman spectroscopy (SERS), which employs metallic surfaces to enhance the Raman signal,^485,486^ have been applied to analytes including proteins, DNA, RNA, cells, and carbonates.^487^ Raman spectroscopy can provide a chemical fingerprint unique to a Raman-active compound and, importantly, on-chip Raman could be applied to microfluidic determination of mineral vs organic content in aerosol samples during INP analysis. Portable or integrated Raman probes and spectrometers have also been developed for microfluidic platforms that can be used for static or continuous flow measurements and are, therefore, amenable to analysis in the field.^488–490^

Continuous on-chip sampling and *in situ* SERS analysis of bioaerosols have been demonstrated by Choi *et al.*^278^ who employed their continuous Dean flow microfluidic impinger^235^ to sample bacteria (*S. epidermidis*, *M. luteus*, *E. hirae*, *B. subtilis*, and *E. coli*). Silver nanoparticles (AgNPs) were introduced into the device in continuous flow, which bound onto the bacterial cells and allowed their identification as they passed through a detection region in the chip, with the quantification of *S. epidermidis* and its monitoring over time also achieved. This demonstrates the possibility for on-chip Raman to be applied to biological INP analysis in future platforms.

### G. Fluorescence spectroscopy

Fluorescence spectroscopy and microscopy are highly sensitive detection tools that are based on the excitation of fluorophore molecules with certain wavelengths of light (excitation), exciting the molecules such that they emit light at a longer wavelength (emission). Direct detection of fluorescent primary biological aerosol particles (FBAPs), including bacteria, pollen, molds, and others, is possible using ultraviolet laser-induced fluorescence (UV-LIF), for example, via online aerosol measurement instruments such as the Wideband Integrated Bioaerosol Sensor (WIBS).^491^ Online UV-LIF measurements^38,56,492,493^ and fluorescence microscopy^56^ have been employed during a number of INP measurement campaigns to compare FBAP concentrations to INP activity.

Fluorescence detection is a common analysis technique in microfluidics, often used in immunoassays and various DNA analyses following the labeling on the target analyte with a fluorophore,^494^ and has been applied to several microfluidic bioaerosol analyses. Kang *et al.*^495^ developed a real-time detection system for bioaerosols using inertial impaction and mini-fluorescent microscopy based on a webcam. A curved channel provided an impaction zone within the microfluidic device, with the particle diameter cutoff determined by the channel dimensions and the flow rate. A camera module from a webcam was combined with filters and a blue light source to observe the FBAPs impacted on the channel wall.

The continuous Dean flow microfluidic impinger developed by Choi *et al.*^235^ was employed for the collection of *S. epidermidis*, followed by its off-chip analysis by fluorescence microscopy. Choi *et al.*^496^ also developed an on-chip flow cytometer for the detection of bioaerosol particles via LIF detection with an integrated optical fiber connected to a photodetector. Samples of *E. coli*, *B. subtilis*, and *S. epidermidis* were collected using a conventional bubble impinger and pumped through the microfluidic chip where they were stained with SYTO82 fluorescent dye and detected as they flowed through the detection region.

### H. Electrical detection

Electrical and electrochemical sensors can be employed for a number of measurements via a range of detection methods (e.g., impedance, voltammetry, and amperometry) and are amenable to integration into microfluidic devices via microfabricated electrodes and SPEs,^497^ while the functionalization of electrodes with antibodies allows for use as sensors for immunoassays.^498,499^ The use of microelectrodes enables small footprint analytical platforms, making electrochemical microfluidic detectors extremely attractive for point-of-care diagnostic devices.^500^ While electrical detection, to our knowledge, has not been applied to INP analysis, electrochemical detection of aerosols has been achieved when using microfabricated systems and microfluidic devices and could be applied to biological INPs in the future.

Kwon *et al.*^245^ incorporated sensing electrodes into the impactor plates of their 3D printed cascade impactor system, allowing for the detection of electrically charged aerosol particles as they were collected on the plates. Yin *et al.*^314^ tested the use of electrical impedance measurements of particles using commercial SPEs, with the intended application to their microfluidic continuous flow DLD platform comprising I-shaped pillars for the separation of PM_2.5_ aerosols. Kim *et al.*^252^ developed a microfabricated single-stage virtual impactor that separated aerosols, whereupon a micro corona discharge was used to charge the separated particles and allow their detection via electrometers based on the electrical current carried by the particles.

### I. Pyroelectric thermal sensors

Pyroelectric materials are capable of generating a voltage when they experience heating or cooling. Cook *et al.*^501^ recently demonstrated that polyvinylidene fluoride (PVDF), an inexpensive pyroelectric polymer that can be purchased in sheets and cut to shape, can be used to detect freezing events during DFAs based on the release of latent heat when a droplet freezes. A sheet of PVDF was placed atop a cold stage and covered in a thin layer of Vaseline, onto which a standard microliter droplet array was pipetted. As the stage was cooled and the droplets froze, the latent heat released by the droplets yielded a spike in voltage in the pyroelectric detection system.

The incorporation of pyroelectric materials, particularly PVDF, into microfluidic devices has been demonstrated for on-chip temperature monitoring via inclusion of a layer of the polymer in the device.^502–504^ Given the wide range of polymer microfabrication methods available,^505–510^ it is conceivable that devices could be manufactured directly out of PVDF if desired.

### J. Infrared thermal imaging

Infrared thermal imaging is another technique that has been used to detect droplet freezing events during DFAs based on the release of latent heat, typically being applied to multiwell plates containing droplet volumes of tens to hundreds of microliters and using a thermal camera.^511–516^ While thermal imaging has not yet been applied to microfluidic INP analysis, it has been employed for monitoring a number of processes (e.g., temperature cycling) in microfluidic devices;^517–521^ hence, it is feasible that it could be used for microfluidic DFAs or for monitoring temperature-dependent biochemical assays.

### K. Differential scanning calorimetry

Differential scanning calorimetry (DSC) also measures the latent heat released upon the freezing of droplets and is capable of very high temperature accuracy. However, it is not capable of detecting individual droplet freezing events as in other methods, typically requiring water-in-oil emulsions for ice nucleation studies. DSC has been applied to a number of ice nucleation studies, including homogeneous freezing, bacterial INPs,^522^ mineral dusts,^523,524^ pollen,^524^ and water confined in silica capsules.^525^

DSC has been applied to microfluidically generated droplet emulsions. Riechers *et al.*^367^ used DSC to demonstrate that absolute temperature is the most important uncertainty in homogeneous freezing measurements, while Lignel *et al.*^368^ tested the stability of droplet emulsions toward experiments in microgravity.

While DSC is not particularly suited to biochemical analysis or for in-the-field monitoring, MEMS-based DSCs have been developed and could be employed for microfluidic ice nucleation studies.^526–530^ The miniaturized dimensions of MEMs technology allow for smaller thermal masses and therefore faster scanning, together with low sample consumption.

## VIII. CHALLENGES AND CONSIDERATIONS

The scope for the application of microfluidic separation and analysis techniques to biological INP measurements is enormous, providing an opportunity to enable the identification and quantification of biogenic INPs as a matter of course for the atmospheric ice nucleation community. However, there are several challenges that must be acknowledged in the development of a sample-to-answer INP platform. Knowledge of these issues allows their consideration or avoidance when building a complex multi-step analysis system.

### A. High density mineral dusts

While biological analysis is the focus here as a means to identify “missing sources” of INPs in models, there must also be a means of assessing the relative contributions of other important INP types such as mineral dusts. This could, for example, be achieved via chemical or spectroscopic (e.g., Raman) analysis, or informed assumptions about the environment that sampling is taking place in and the sources of the air masses that have been sampled. Parallelized screening with chemical treatment and repeat DFAs could allow for categorization or classification of the organic vs inorganic components.

However, an issue with relatively large (several micrometers in diameter) mineral dust particles, which have a high density (∼2.65 g cm^−3^), is that they can sediment relatively quickly (e.g., 90 *μ*m s^−1^ for a 10 *μ*m diameter K-feldspar particle). This can potentially be problematic when using syringes and pumps to drive them through a microfluidic device.^201^ Such an issue could be alleviated, however, via the use of perpetual sedimentation pumps,^531^ in-syringe magnetic stirrer bars (including commercial products such as the Cetoni Nemix 50),^532^ or the incorporation of magnetic stirrer bars into reservoirs used in pressure-based systems (e.g., pressure controllers from Dolomite, Elveflow, or Fluigent).

### B. Sampling and analysis of rare INPs and bioaerosols

INPs are an important but incredibly rare subset of aerosols, often comprising only 1 in 10^3^–10^6^ ambient particles in the troposphere.^4^ The most active INPs (i.e., those that trigger freezing at warmer temperatures), such as biogenic species, are also the rarest but can have a great impact, while the less active particles can have much larger concentrations. Therefore, to capture the rarer but important warm-temperature INPs, it can be necessary to sample hundreds or even thousands of liters of air to obtain INP signals above an instrument's detection limit once the particles have been washed into or collected as an aqueous suspension.

The problem is further compounded by the volume of aqueous suspension that is processed in a microfluidic device. If 50 droplets of 1 *μ*l volume are analyzed in a conventional microliter DFA, then nearly 100 000 droplets of 100 *μ*m diameter (∼524 pl) would need to be analyzed in a microfluidic DFA in order to process the same volume of sample, thereby ensuring that the rarer particles detected in the former are also captured in the latter. It is for this reason that many microfluidic DFA results show INP concentrations in a colder temperature regime than standard microliter assays. This is nonetheless useful to access temperature regimes that standard microliter DFAs cannot, but by greatly increasing the droplet throughput and automation of microfluidic DFAs it is highly feasible that INP concentrations could be obtained across the entire relevant temperature spectrum (around −35 to 0 °C). This may be where continuous flow DFAs, which would also be more amenable to upstream and downstream processing techniques, come to the fore in order to easily analyze tens or hundreds of thousands of droplets.

Therefore, an ideal system would combine high throughput air sampling with small liquid collection volumes to greatly increase aerosol concentrations for analysis. This is where direct sampling into a microfluidic device with a form of sample concentration would be of great benefit. A further issue for bioaerosol analysis, in general, can be the scarcity of the target analytes compared to background contaminants that can interfere with the detection of bioaerosols, resulting in false negatives or artificially low results.

### C. Microfluidic DFA analysis

While microfluidic DFAs can enable the processing of thousands of droplets in DFAs, this could present an issue in automated platforms in terms of their analysis. Currently, microfluidic DFAs employ cameras to observe droplet freezing events, with videos analyzed either manually or using an automated program on an experiment-by-experiment basis. In the case of continuous flow DFAs, high-speed cameras are required that generate a large amount of data and whose analyses are more difficult to automate. This could be alleviated by the use of machine learning-based analysis,^206^ for example, while modification of droplet throughput could enable the use of cameras that do not need to operate at high speed.

A far more suitable method for analysis in a sample-to-answer platform would be to remove the camera entirely and replace it with a single-point detection system that provides a readout of a detection signal over time. On-chip laser light scattering^533^ may allow for frozen and unfrozen droplets to be distinguished using a single detection system. Alternatively, the use of a droplet sorting system to separate frozen and unfrozen droplets into separate outlets with 100% efficiency would allow for simple light-based or electrochemical detectors to detect the number of droplets that pass through each channel. This would greatly simplify the output and analysis of DFAs, particularly when processing tens of thousands of droplets.

A recent consideration that affects many of the microfluidic DFA techniques discussed here is the discontinuation of many of the per- and polyfluoroalkyl substances (PFAS) (e.g., Fluorinert^™^ FC-40, Novec^™^ 7500) that are used as the immiscible oil in droplet production. These oils provide excellent heat transfer properties, low pour points, and both hydrophobic and oleophobic properties that make them ideal for DFAs. However, they are highly persistent, allowing these “forever chemicals” to accumulate in organisms and the environment, and have implications for human health and ecology.^534^ Therefore, they have started being phased out of production since they will likely start to become significantly regulated and restricted in the near future,^535,536^ though the impact on their use in microfluidics may be less immediate.^537^ Therefore, other less harmful and persistent PFAS-free oils with suitable properties for the DFA of choice will need to be employed in the future.

Thankfully, several microfluidic DFAs, particularly array-based DFAs, already use PFAS-free oil and surfactant systems (see Table II), with some such as the “store and create,”^204,388^ printed array,^384^ or microcavity^321^ methods not requiring surfactant (or even oil in some cases).^383,396^ The issue may impact the droplet emulsion (in terms of emulsion stability) and continuous flow DFAs (in terms of the oil viscosity at colder temperatures) the most, but there are a wide variety of oil-surfactant systems that may be suitable (see Hauptmann *et al.*^353^ and Baret,^538^ for example) while PFAS-free oils with similar properties to PFASs will also likely be developed as a replacement. The discontinuation of PFASs affects not only DFAs but microfluidic droplet applications, in general, given their widespread use for forming highly stable droplets,^539,540^ which should facilitate the discovery of a suitable alternative in the shorter term given the common goal of the community.^537^

### D. Reagent storage

As discussed earlier (in Sec. VII C), one of the considerations for long term bioaerosol monitoring or analysis in-the-field is the stability of the reagents. Some reagents may have a short lifetime, particularly if not properly stored or when prepared as an aqueous solution. This remains a consideration in fields such as point-of-care clinical diagnostics, but for this reason there have been a myriad of solutions to microfluidic reagent storage and release developed over the years for bioanalytical purposes.^541^ These include the use of lyophilized reagents in reservoirs or spotted into microfluidic channels that can be reconstituted as and when required. Liquid reagents can be held in blister packs prior to their mixing for a reaction, while the implementation of various micropump and microvalve techniques can allow for chemicals to be accessed and released at specific timeframes.

### E. Integration of components and processes

This review has demonstrated that there are a myriad of microfluidic techniques available for each individual step of an envisaged sample-to-answer INP bioanalysis platform, with each offering a range of operational conditions and benefits or drawbacks. However, particularly for bioaerosol analysis, only a handful of examples thus far exist in which several of these steps have been integrated together.^216,237,238,287–289^

Various operating parameters and compromises must be considered when integrating multiple components, since some may function in very different regimes to others. This may be in terms of flow rates, throughput, temperatures, and particle sizes. Sample carry-over and biofouling can also be issues in sample-to-answer monitoring systems, hence rigorous cleaning processes may be required between samples, and the use of single-shot consumables.

While the potential for complex integrated systems is enormous and feasible, it may often be easier and faster to remove some functionality in order to produce an integrated platform that is less complex but more robust and reliable in performing a specific purpose. The thoughtful selection of compatible techniques for integration is important, and compromise is key.

## IX. CONCLUSIONS

Microfluidics has the potential to revolutionize biological INP analysis by providing a toolbox of bioanalytical separation and analysis techniques that have been developed over decades for point-of-care diagnostics and medical applications. These proven capabilities, combined with miniaturized aerosol sampling technologies and microfluidic droplet freezing assays (DFAs) that have been in development for over a decade and allow high droplet number DFAs down to homogeneous freezing, provide an opportunity to produce novel, small footprint, sample-to-answer platforms that could be deployed in the field for automated and even remote sensing of atmospheric INPs.

This has the potential for the construction of a network of micro total analysis systems (*μ*TAS) that would enable continuous measurement of atmospheric INPs at monitoring stations around the world, providing unprecedented data sets describing the spatial and temporal behavior of INPs in terms of their concentrations and composition. Such an endeavor would greatly improve our understanding of atmospheric INPs and enable better representation in global models, in turn reducing the uncertainties in aerosol-cloud interactions and climate projections.

However, this is not without its challenges. While there are many possible methodologies available for performing each step of the sample-to-answer process, not all are compatible with each other, and even those that are will likely face fluid and mechanical engineering challenges related to integration of different procedures, e.g., flow rates, timings, reagent lifetimes, and compatibilities. Nonetheless, the necessary tools are already in place to achieve this, and overcoming these challenges will pave the way for a revolutionary atmospheric INP analysis platform that will dramatically enhance our ability to predict and understand the impacts of a changing climate.

## SUPPLEMENTARY MATERIAL

Tables I and II from the Appendix are available as supplementary material in the form of downloadable CSV files. The CSV of Table I contains a comprehensive list of known biological INPs. The CSV of Table II provides details of microfluidic droplet freezing assays, including their operating parameters and the types of samples that have been processed.

## Data Availability

Data sharing is not applicable to this article as no new data were created or analyzed in this study.

## APPENDIX: BIOLOGICAL ICE-NUCLEATING PARTICLES AND MICROFLUIDIC DROPLET FREEZING ASSAYS

Table I provides a list of known biological ice-nucleating particles and Table II provides a list of microfluidic droplet freezing assays (DFAs), their operating parameters, and samples that have been analyzed.
